# Effects of the Haemodynamic Stimulus on the Location of Carotid Plaques Based on a Patient-Specific Mechanobiological Plaque Atheroma Formation Model

**DOI:** 10.3389/fbioe.2021.690685

**Published:** 2021-06-14

**Authors:** Patricia Hernández-López, Myriam Cilla, Miguel Martínez, Estefanía Peña

**Affiliations:** ^1^Aragón Institute of Engineering Research (I3A), University of Zaragoza, Zaragoza, Spain; ^2^Centro Universitario de la Defensa, Academia General Militar, Zaragoza, Spain; ^3^Biomedical Research Networking Center in Bioengineering, Biomaterials and Nanomedicina (CIBER-BBN), Zaragoza, Spain

**Keywords:** atheroma plaques, atherosclerosis, carotid artery, convection-diffusion-reaction equations, mechanical stimulus, mechanobiological model, patient-specific

## Abstract

In this work, we propose a mechanobiological atheroma growth model modulated by a new haemodynamic stimulus. To test this model, we analyse the development of atheroma plaques in patient-specific bifurcations of carotid arteries for a total time of 30 years. In particular, eight geometries (left or right carotid arteries) were segmented from clinical images and compared with the solutions obtained computationally to validate the model. The influence of some haemodynamical stimuli on the location and size of plaques is also studied. Plaques predicted by the mechanobiological models using the time average wall shear stress (TAWSS), the oscillatory shear index (OSI) and a new index proposed in this work are compared. The new index predicts the shape index of the endothelial cells as a combination of TAWSS and OSI values and was fitted using data from the literature. The mechanobiological model represents an evolution of the one previously proposed by the authors. This model uses Navier-Stokes equations to simulate blood flow along the lumen in the transient mode. It also employs Darcy's law and Kedem-Katchalsky equations for plasma and substance flow across the endothelium using the three-pore model. The mass balances of all the substances that have been considered in the model are implemented by convection-diffusion-reaction equations, and finally the growth of the plaques has been computed. The results show that by using the new mechanical stimulus proposed in this study, prediction of plaques is, in most cases, better than only using TAWSS or OSI with a minimal and maximal errors on stenosis ratio of 2.77 and 32.89 %, respectively. However, there are a few geometries in which haemodynamics cannot predict the location of plaques, and other biological or genetic factors would be more relevant than haemodynamics. In particular, the model predicts correctly eleven of the fourteen plaques presented in all the geometries considered. Additionally, a healthy geometry has been computed to check that plaque is not developed with the model in this case.

## 1. Introduction

Atherosclerosis is a disease that causes the formation of atheroma plaques in arterial walls. The effect of atheroma plaques is that the thickness of the arterial wall increases-due to an accumulation of some substances such as low density lipoproteins (LDL) and foam cells (FC) in it-and, therefore, the lumen area decreases and blood cannot flow properly. It can derive in several events, such as heart attacks, ischaemias or strokes, and currently it is one of the main causes of mortality in developed countries (Gaziano and Gaziano, 2012). Although this pathology has been widely studied, it has not yet been completely understood. Therefore, it is relevant to study the process of formation of atheroma plaques and to foresee the locations that are susceptible to the emergence of plaques in arteries.

It has been accepted that some mechanical stimuli can cause shape changes of endothelial cells (Dai et al., 2004), and depending on it, they can induce LDL transport into the arterial wall through the endothelium, initiating the growth of atheroma plaques in the vessel. These mechanical stimuli can depend on several factors such as cyclic stretches, cardiac cycle, geometry of the arteries and oscillatory shear stress (Ohayon et al., 2011).

One of these mechanical stimuli is the wall shear stress (WSS) caused by blood flow in the endothelium. It is an index that has been widely used to predict the location of plaques; however, it has the limitation of being calculated for stationary blood flow and does not consider the cardiac cycle. It is well known that areas with physiological WSS promote endothelial cells to have an elongated shape, so pores between them are small and limit the flow of substances across the endothelium. In contrast, for areas with very low WSS, endothelial cells are more circular, so pores are larger and allow flow of substances between them, resulting in plaque emergence. The threshold of WSS below which plaques grow depends on the considered artery (Olgac et al., 2008; Filipovic et al., 2013). In the case of carotid arteries, areas with WSS lower than 2 Pa could be considered atheroprones, while areas of higher WSS are atheroprotectives (Zhao et al., 2002; Younis et al., 2004; Filipovic et al., 2013).

To avoid the limitation of not considering transient blood flow, other studies use the time averaged wall shear stress (TAWSS) instead of WSS to take into account the cardiac cycle and to improve the accuracy of the prediction (Sáez et al., 2015; Alimohammadi et al., 2017). Another index that has also been used in some studies is the oscillatory shear index (OSI). There is some evidence about the influence of this mechanical stimulus on cell shape, and therefore in the emergence of plaques, being areas of high OSI susceptible to developing plaques (Alimohammadi et al., 2017). This index also considers the complete cardiac cycle. However, most of these studies only take into account TAWSS or OSI to predict the location of plaques and do not consider the inflammatory process to reproduce the growth of plaques.

There are other indices that have been investigated recently such as Cross-flow index (CFI) (Arshad et al., 2020), Transverse Wall Shear Stress (transWSS) (Peiffer et al., 2013) and Topological Shear Variation Index (TSVI) (Morbiducci et al., 2020), but their implementation into the model was not possible due to that there are not enough experimental data correlating SI of endothelial cells with them (Morbiducci et al., 2020).

Finally, some studies use patient-specific geometries and calculate blood flow in the transient mode with growth of plaques, but they do not consider all the substances that take part in the disease progression and do not add volume to the final plaque (Filipovic et al., 2013; Díaz-Zuccarini et al., 2014; Alimohammadi et al., 2017).

In this study we use patient-specific geometries with different degrees of atheroma plaques in carotid arteries to fictitiously reconstruct healthy arteries and to computationally reproduce the inflammatory process of the emergence of plaques in them. We use transient blood flow, taking into account the cardiac cycle, and analyse plaque growth under three different hypotheses considering distinct mechanical stimuli: TAWSS, OSI, and a combination of them that we propose as a new stimulus.

The aim of this study is to analyse the predictability of the different mechanical stimuli to estimate the emergence of plaques, improving a previous model developed by the authors under an axisymmetric hypothesis (Cilla et al., 2014). Finally, we compare the plaques predicted using the computational mechanobiological model with the real plaques of patients in clinical images in order to determine which haemodynamical stimulus can better predict the location and size of plaques.

## 2. Materials and Methods

The mechanobiological model proposed by Cilla et al. (2014) was improved by the introduction of a new mechanical stimulus and some terms were simplified in order to improve the numerical convergence, see section 2.5. The endothelium was modelled as a thin layer of endothelial cells, which change their shape as a function of the haemodynamical stimulus -TAWSS, OSI and a combination of them- by becoming rounder or elongated according to the stimulus and thus allowing more or less substance transport along the endothelium. The arterial wall was modelled as a single layer (intima-media) with a permeable membrane (endothelium).

### 2.1. Patient-Specific Geometries

**Figure 1:**
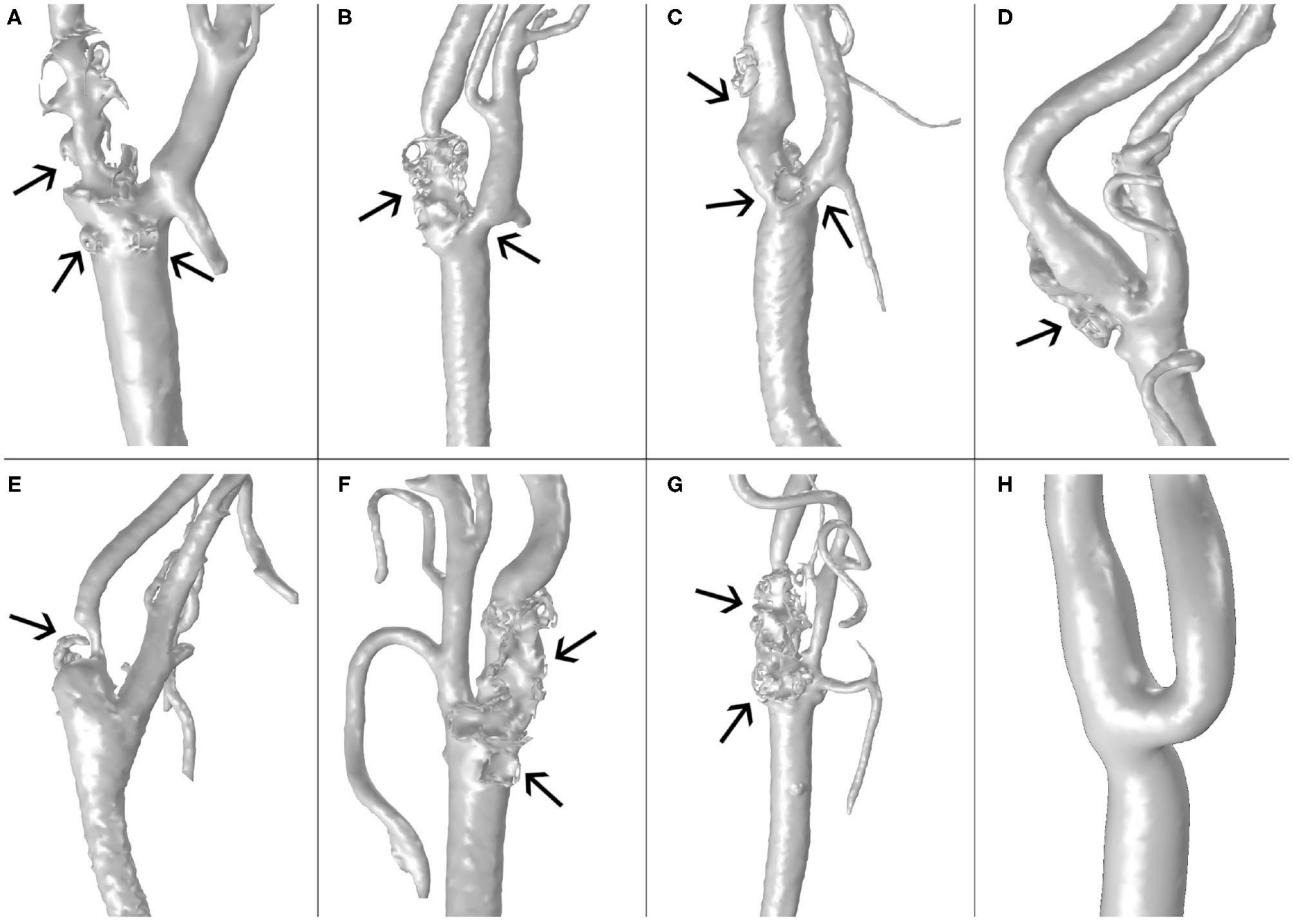
Images the carotid geometries of all patients with atheroma plaque **(A–G)** and the healthy volunteer **(H)**.

Clinical images of four different male patients with atherosclerosis and one healthy volunteer were segmented using the software Materialise Mimics (Materialise N. V., Leuven, Belgium) to obtain eight different patient-specific geometries of carotid artery bifurcations, including common, internal and external carotid arteries (CCA, ICA and ECA, respectively). The clinical images were provided by the Hospital Clinico Universitario in Zaragoza, Spain, according to ethics guidelines of the hospital. One geometry corresponds to a healthy volunteer without pathology, and the others correspond to four patients with developed atheroma disease. Carotid images are shown in Figure 1, with their respective plaques indicated by arrows. The images in Figure 1 correspond to the real geometries of the patients, without making changes in them, to show the differences between the real carotids and the computed ones, that can be observed in **Figure 7**. These differences are due to the simplifications that are necessary to compute the model, e.g., the small side branches of the carotids were eliminated in the computed geometry to simplify the model and it could have some influence on the blood flow distribution.

A thresholding segmentation technique was used to reconstruct the lumen of the vessel from the clinical images. Once the thresholding was done, the plaques of the carotids were located, and plaques were digitally removed to obtain geometries that we considered healthy arteries previous to the development of the pathology. The geometry corresponding to the healthy volunteer was not modified. Finally, the arterial wall was extruded with the software Rhinoceros (Robert McNeel & Associates, Seattle, WA, United States) from the lumen to obtain a 3D geometry with variable thickness, imposing a thickness of 0.7 mm for the CCA and 0.53 mm to the ICA and ECA. Finally, the thickness in the area close to the bifurcation was progressively reduced when advancing from the CCA to de ICA and ECA according to their respective thicknesses (Sommer et al., 2010).

The different geometries were coded from “A” to “H.” The patient-specific geometry “A” was used to estimate the parameters not found in the literature to computationally reproduce the location and size of the real plaque. Once done these estimations, these parameters were used for the rest of geometries. The healthy volunteer, named “H,” was used to demonstrate that the mechanobiology model can also predict a healthy case without relevant growth of plaques. The total time of the numerical simulations implemented was 30 years (Insull, 2009) and patients were supposed to have a high level of hypercholesterolemia with an LDL concentration in blood of 6.98 $\frac{mol}{m^{3}}$, equivalent to 270 $\frac{mg}{dL}$ (Goldstein and Brown, 1977).

### 2.2. Numerical Methods

The geometries were meshed using triangular elements. Mesh sensitivity analysis was performed for both the lumen and the arterial wall, including the number of boundary layers, to determine optimal meshes to finally compute the whole process. The final mesh had a total of 850,000 elements for the lumen, with two boundary layers near the endothelium, and 550,000 elements for the arterial wall, with boundary layers near the endothelium and the adventitia to ensure the correct calculation of fluxes across the arterial wall.

**Figure 2:**
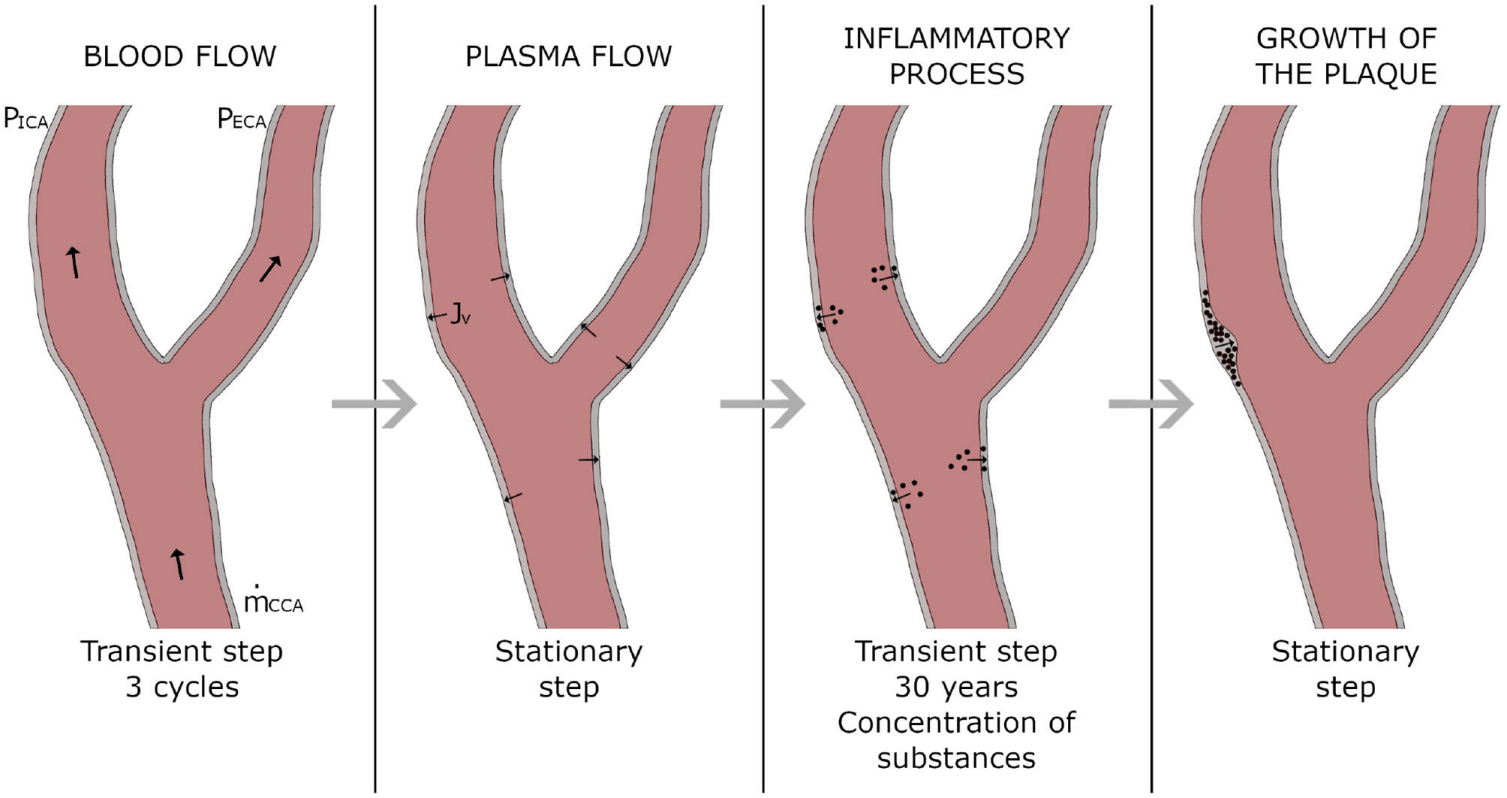
Workflow of the computational process.

The software COMSOL Multiphysics (COMSOL AB, Burlington, MA, USA) was used to computationally solve the model following four consecutive stationary and transient steps. A first transient step was used to simulate the blood flow along three cardiac cycles. Then, a second step under stationary hypothesis was performed to solve the plasma flow across the endothelium. Afterwards a third step was computed in transient mode to calculate the concentrations of all the substances in all the arterial wall during 30 years, and finally, a fourth and last stationary step was made to compute the growth of the plaques from the concentration of all the substances at 30 years. In Figure 2 there is a scheme with the followed workflow.

A direct linear solver (PARDISO) was used to solve the transient blood flow along the lumen. Another direct linear solver (MUMPS) was employed to compute the plasma flow through the endothelium, the inflammatory process of all the substances (in an iterative way, using different segregated steps for groups of substances) and finally the growth of the plaques.

The development of the mathematical model is presented in the following sections, separating the equations referring to the blood flow, plasma flow, inflammatory process and growth of plaques.

### 2.3. Blood Flow Model

According to Caro et al. (1978) and Perktold et al. (1991), blood was modelled as a Newtonian and incompressible fluid because the lumen diameter of the considered arteries is higher than 0.5 mm. Additionally, blood flow was considered laminar due to the Reynolds number in carotid arteries under physiological conditions. Blood is basically composed of a liquid component called plasma, but it also contains solid particles. Nevertheless, these particles are very small in comparison to the lumen diameter. Therefore, blood was considered a homogeneous fluid (Malvè et al., 2014).

Blood flow in the lumen is governed by Navier-Stokes and continuity equations:

(1) $$\begin{array}{l} \rho_{b}\left(u_{l}\cdot \nabla \right)u_{l}=\nabla \cdot \left[-P_{l}I+\mu_{b}\left(\nabla u_{l}+{\left(\nabla u_{l}\right)}^{T}\right)\right]+F_{l} \end{array}$$

(2) $$\begin{array}{l} \rho_{b}\nabla \cdot u_{l}=0, \end{array}$$

where subscripts *b* and *l* refer to blood and lumen, respectively, so parameters ρ_*b*_ and μ_*b*_ are the density and dynamic viscosity of blood, respectively, while *u*_*l*_ and *P*_*l*_ are the velocity and pressure of blood flow in the lumen, respectively. Finally, *F*_*l*_ corresponds to internal forces of the fluid, which are negligible in comparison with the friction between blood flow and the arterial wall. All parameters necessary to calculate blood flow along the lumen are shown in Table 1.

**Table 1:** List of parameters necessary to calculate blood flow along the lumen.

**Blood flow parameters**			
**Parameter**	**Description**	**Value**	**Reference**
ρ_*b*_	Blood density	$1050\frac{kg}{m^{3}}$	Milnor, 1989
μ_*b*_	Blood viscosity	0.0035 *Pa* · *s*	Milnor, 1989
*T*	Cardiac cycle period	0.85 *s*	Malvè et al., 2014

**Figure 3:**
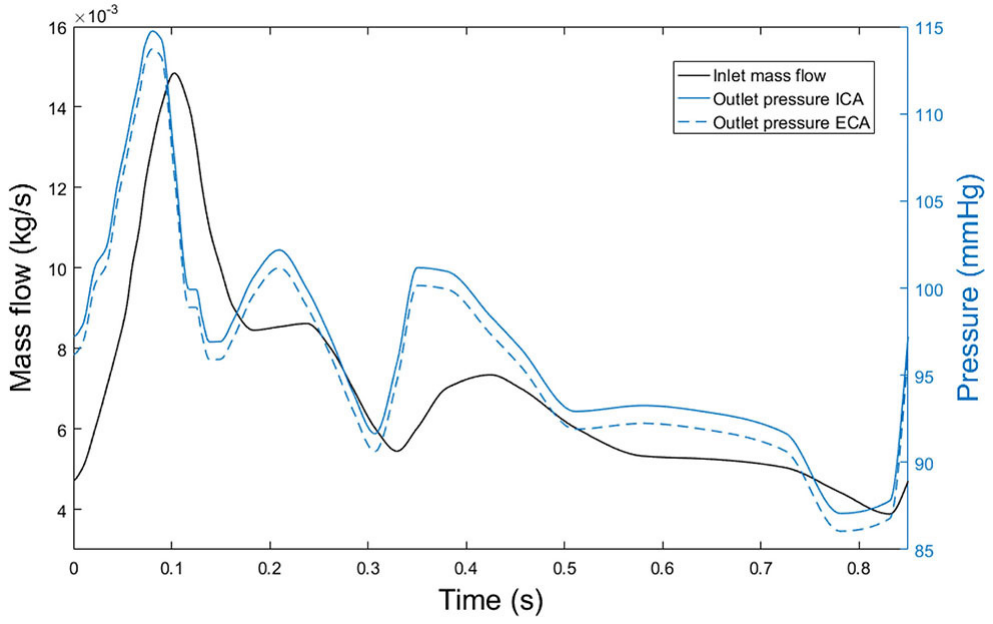
Blood mass flow at the inlet of the CCA of the first patient (black line) and pressure at outlets of both ICA and ECA, (blue continuous and blue dashed lines, respectively).

Blood flow was modelled in the transient mode. Therefore, an analysis of the number of cardiac cycles necessary to model blood flow was performed, obtaining that a total number of three cycles is sufficient to completely develop blood flow and establishing the validity of the results of flow obtained for the third cardiac cycle. At the inlet of the lumen, transient mass flow was imposed, and transient pressures were imposed at the two outlets of the lumen. Transient flow and pressures were imposed following their respective shapes along a cardiac cycle obtained from Malvè et al. (2014). Additionally, Murray's law was applied in all the geometries to establish the correct division of blood flow at the bifurcations, with an average pressure at the outlet of the ICA of 100 mmHg. In Figure 3, blood flow at the inlet of the CCA and pressures at the outlets of ICA and ECA imposed at geometry A can be seen. Finally, a non-slip condition was imposed at the endothelium.

### 2.4. Plasma Flow Across the Endothelium

The arterial wall is permeable; therefore, some elements contained in the blood flow can cross the arterial solid wall. In particular, there is a plasma flow through the endothelium from the lumen that can be modelled with Darcy's Law:

(3) $$\begin{array}{l} u_{w}=\frac{k_{w}}{\mu_{p}}\nabla p_{w}, \end{array}$$

where *u*_*w*_ and *k*_*w*_ are the velocity of the plasma on the arterial wall and its permeability, respectively. ∇*p*_*w*_ is the pressure gradient in the arterial wall, and finally, μ_*p*_ is the dynamic viscosity of plasma.

Furthermore, continuity of plasma flow has to be accomplished:

(4) $$\begin{array}{l} \frac{\partial \left(\rho_{p}\epsilon_{w}\right)}{\partial t}+\rho_{p}\nabla \cdot u_{w}=J_{v}, \end{array}$$

where ρ_*p*_ and ϵ_*w*_ are the density of plasma and the porosity of the arterial wall, respectively. The last term, denoted as *J*_*v*_, is the plasma flow through the endothelium. It can be calculated with Kedem-Katchalsky equations, considering three different types of pores in the endothelium by which plasma flow is allowed: normal junctions, leaky junctions and vesicular pathways. However, plasma flow through vesicular pathways is very small related to the other two, so it is negligible (Olgac et al., 2008). Total plasma flow throughout the endothelium (*J*_*v*_) can be calculated as:

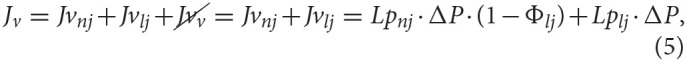



where *Jv*_*nj*_, *Jv*_*lj*_, and *Jv*_*v*_ are plasma flows across normal junctions, leaky junctions and vesicular pathways, respectively. Finally, *Lp*_*nj*_ and *Lp*_*lj*_ are the hydraulic conductivities of normal and leaky junctions, respectively. The value of *Lp*_*nj*_ depends on the thickness of the arterial wall (and, therefore, of the artery that we consider) and of the intraluminal pressure (Tedgui and Lever, 1984). Δ*P* is the pressure drop in the endothelium, which depends on the intraluminal pressure and takes values of 18 and 28 mmHg for an intraluminal pressure of 70 and 180 mmHg, respectively, Tedgui and Lever (1984). Finally, Φ_*lj*_ is the fraction of leaky junctions and is defined as the ratio of the number of leaky cells and the total number of cells (Weinbaum et al., 1985; Huang et al., 1994; Huang and Tarbell, 1997), and in our model, it depends on the haemodynamical stimulus and the number of mitotic cells, see section 2.6.

Following Weinbaum et al. (1985) and Yuan et al. (1991), hydraulic conductivity of leaky junctions can be calculated as:

(6) $$\begin{array}{l} Lp_{lj}=\frac{Ap}{S}\cdot Lp_{slj}=\frac{4w_{l}}{R_{cell}}\cdot \Phi_{lj}\cdot Lp_{slj}, \end{array}$$

where $\frac{Ap}{S}$ is the fraction of total area occupied by leaky junctions, *Lp*_*slj*_ is the hydraulic conductivity of a single leaky junction, *R*_*cell*_ the radius of endothelial cells, and *w*_*l*_ the half-width of a leaky junction (Weinbaum et al., 1985; Yuan et al., 1991; Huang et al., 1994). For more details about the derivation of (6), see Appendix A.

The total number of mitotic cells in the endothelium depends on the haemodynamical stimulus that we are using. In experimental studies, the number of mitotic cells (MC) was determined in areas of the known shape index (SI) (Chien, 2003), so it was developed in the next experimental correlation with a unit area of 0.64 *mm*^2^:

(7) $$\begin{array}{l} MC=0.003797\cdot e^{\left(14.75\cdot SI\right)} \end{array}$$

In addition, based on experimental studies, the next correlation between the number of leaky cells (LC) and mitotic cells for the unit area of 0.64*mm*^2^ is defined (Lin et al., 1989; Olgac et al., 2008):

(8) $$\begin{array}{l} LC=0.307+\frac{0.805\cdot MC}{0.453} \end{array}$$

Φ_*lj*_ is defined as the ratio between the area of leaky cells and the area of all the cells, and it can be calculated as:

(9) $$\begin{array}{l} \Phi_{lj}=\frac{LC\cdot \pi \cdot R_{cell}^{2}}{A_{unit}}, \end{array}$$

taking as *A*_*unit*_ the unit area considered in all the anterior experimental correlations of 0.64 *mm*^2^. By using all the experimental correlations, it is now possible to obtain Φ_*lj*_ and, therefore, $\frac{A_{p}}{S}$. Consequently, knowing the SI that it is computed by Equations [(32)–(35)] for our model, we can compute Φ_*lj*_ and the hydraulic conductivity *Lp*_*lj*_.

Finally, the hydraulic conductivity of a unique leaky junction, *Lp*_*slj*_, is defined following Olgac et al. (2008):

(10) $$\begin{array}{l} Lp_{slj}=\frac{w_{l}^{2}}{3\cdot \mu_{p}\cdot l_{lj}}, \end{array}$$

where μ_*p*_ is the dynamic viscosity of the plasma, and *w*_*l*_ and *l*_*lj*_ the width and the length of a leaky junction, respectively. Therefore, with this derivation, plasma flow through the endothelium is completely determined. The parameters necessary for the plasma flow calculation are depicted in Table 2.

**Table 2:** List of parameters necessary to compute plasma flow across the endothelium.

**Plasma flow parameters**			
**Parameter**	**Description**	**Value**	**Reference**
*R*_*cell*_	Endothelial cell radius	15 μ*m*	Weinbaum et al., 1985
*w*_*l*_	Half-width of a leaky junction	20 *nm*	Weinbaum et al., 1985
*l*_*lj*_	Leaky junction length	2 μ*m*	Weinbaum et al., 1985
ρ_*p*_	Plasma density	$1050\frac{kg}{m^{3}}$	Milnor, 1989
μ_*p*_	Plasma viscosity	0.001 *Pa* · *s*	Milnor, 1989
*k*_*w*_	Darcian artery permeability	1.2 · 10^−18^ *m*^2^	Vargas et al., 1979
ϵ_*p*_	Intima porosity	0.96	Ai and Vafai, 2006
*L*_*p,nj*_	Normal junction conductivity	$1.984\cdot 10^{-12}\frac{m}{s\cdot Pa}$	Tedgui and Lever, 1984
Δ*P*_*r*_	Endothelial pressure difference	20.727 *mmHg*	Tedgui and Lever, 1984
*A*_*unit*_	Unit area for the experimental correlations	0.64 *mm*^2^	Chien, 2003
*P*_*adv*_	Pressure of the adventitia	17.5 *mmHg*	Olgac et al., 2008

The normal velocity of plasma flow through the endothelium is *J*_*v*_, which has already been defined in section 2.4. Additionally, the pressure at adventitia defined in Olgac et al. (2008) is also prescribed (17.5 mmHg).

### 2.5. Inflammatory Process of the Arterial Wall

Once plasma flow across the endothelium has been modelled, we can compute the inflammatory process that takes place on the arterial wall. There are many substances involved in this process, among which we consider LDL, oxidized LDL (LDLox), monocytes (m), macrophages (M), cytokines (C), contractile and synthetic smooth muscle cells (CSMC and SSMC), foam cells (FC), and collagen (G).

The behaviour of cells and substances on the arterial wall obeys convection-diffusion-reaction equations of the form:

(11) $$\begin{array}{l} \underset{time}{\underset{︸}{\frac{\partial X_{i}}{\partial t}}}+\underset{diffusion}{\underset{︸}{\nabla \cdot \left(-D_{X_{i}}\nabla X_{i}\right)}}+\underset{convection}{\underset{︸}{K_{lag}\cdot u_{w}\cdot \nabla X_{i}}}=\underset{source-sink}{\underset{︸}{f_{Xi}\left(\cdots \;,X_{i},\cdots \;\right)}}, \end{array}$$

where *X*_*i*_ is the concentration of the considered substance and *D*_*X*_*i*__ its diffusion coefficient on the arterial wall. *K*_*lag*_ is the solute lag coefficient. The first term of the equation corresponds to temporal variations of the cells or the substances on the arterial wall, while second and third terms are, respectively, diffusion and convection of the cells or substances. Finally, the reaction term represents the interaction between cells and/or substances (chemotaxis, proliferation, differentiation, apoptosis, degradation or generation), and they are different for each of the considered cells or substances. All the parameters for the inflammatory process are collected in Table 3.

**Table 3:** List of parameters necessary to calculate the inflammatory process on the arterial wall.

**Inflammatory process parameters**			
**Parameter**	**Description**	**Value**	**Reference**
*D*_*LDL,w*_	LDL	$8\cdot 10^{-13}\frac{m^{2}}{s}$	Prosi et al., 2005
*D*_*m,w*_	Monocytes	$8\cdot 10^{-15}\frac{m^{2}}{s}$	Budu-Grajdeanu et al., 2008
*D*_*LDLox,w*_	Oxidized LDL	$8\cdot 10^{-13}\frac{m^{2}}{s}$	Prosi et al., 2005
*D*_*M,w*_	Macrophages	$1\cdot 10^{-15}\frac{m^{2}}{s}$	Budu-Grajdeanu et al., 2008
*d*_*LDL*_	LDL oxidation	2.85 · 10^−4^ *s*^−1^	Ai and Vafai, 2006
*d*_*m*_	Monocyte differentiation	1.15 · 10^−6^ *s*^−1^	Bulelzai and Dubbeldam, 2012
*m*_*d*_	Monocyte natural death	$\frac{1}{60}d^{-1}$	Bulelzai and Dubbeldam, 2012
*LDL*_*ox,r*_	Oxidized LDL uptake	$2.45\cdot 10^{-23}\frac{m^{3}}{cells\cdot s}$	Zhao et al., 2006
*n*_*FC*_	Maximum oxidized LDL uptake	$2.72\cdot 10^{-11}\frac{mol}{cells}$	Estimated
*C*_*r*_	Cytokine production	$3\cdot 10^{-10}\frac{m^{3}}{cells\cdot s}$	Siogkas et al., 2011
*d*_*c*_	Cytokine degradation	2.3148 · 10^−5^ *s*^−1^	Zhao et al., 2005
*S*_*r*_	CSMC differentiation	0.0036 *s*^−1^	Chamley-Campbell et al., 1981
*p*_*ss*_	SSMC proliferation	0.24 *d*^−1^	Boyle et al., 2011
*G*_*r*_	Collagen production	$2.472\cdot 10^{-21}\frac{kg}{cells\cdot s}$	Zahedmanesh et al., 2012
*d*_*G*_	Collagen degradation	$\frac{1}{30}d^{-1}$	Humphrey, 2002
*w*_*l*_	Half-width of a leaky junction	20 *nm*	Weinbaum et al., 1985
*l*_*lj*_	Leaky junction length	2 μ*m*	Weinbaum et al., 1985
*R*_*LDL*_	LDL radius	11 *nm*	Prosi et al., 2005
$C_{m,w}^{th}$	Monocyte mitosis	$550\cdot 10^{9}\frac{cells}{m^{3}}$	Khan, 2009
$C_{c,w}^{th}$	Cytokines	$1.235\cdot 10^{13}\frac{mol}{m^{3}}$	Estimated
$C_{SSMC,w}^{th}$	Smooth muscle cells	$4.764\cdot 10^{13}\frac{cells}{m^{3}}$	Boyle et al., 2011
*C*_0, *LDL*_	LDL initial concentration	$6.98\frac{mol}{m^{3}}$	Schwenke and Carew, 1989
*C*_0, *m*_	Monocyte initial concentration	$550\cdot 10^{9}\frac{cells}{m^{3}}$	Khan, 2009
*C*_0, *CSMC*_	CSMC initial concentration	$3.16\cdot 10^{13}\frac{cells}{m^{3}}$	Boyle et al., 2011
*TAWSS*_0_	Reference TAWSS	1 *Pa*	Estimated
*C*_*LDL,adv*_	LDL concentration at adventitia	11.6‰ · *C*_*LDL,l*_	Meyer et al., 1996
*k*_*c*_	Cytokine threshold factor	0.65093	Estimated
*LDL*_*dep*_	LDL deposited at the endothelium	$10^{-2}\cdot C_{LDL,l}$	Meyer et al., 1996
*m*_*r*_	Monocyte recruitment	$6.636\cdot 10^{-4}\frac{m^{4}}{mol\cdot day}$	Steinberg et al., 1997
*r*_*apop*_	SSMCs apoptosis rate	0.087 *s*^−1^	Bennett et al., 1995
ρ_*LDL*_	LDL density	$1063\frac{kg}{m^{3}}$	Ivanova et al., 2017
*Mw*_*LDL*_	LDL molecular weight	$386.65\frac{g}{mol}$	Guarino et al., 2006
*k*_*lag*_	Solute lag coefficient of LDL	0.893	Dabagh et al., 2009

On the other hand, flow of substances across the arterial wall can be defined as:

(12) $$\begin{array}{l} N=-D_{X_{i}}\nabla X_{i}+u_{w}X_{i} \end{array}$$

Initial concentrations of all the substances at the artery wall are null, except for the case of CSMCs, by which all of the arterial wall is composed at the beginning of the inflammatory process. In addition, we suppose a hypercholesterolemia level for all the patients, and their concentrations of LDL and monocytes at the lumen are *C*_*LDL,l*_ and *C*_*m,l*_, respectively.

The specification of these equations for each of the substances and cells of the process is as follows.

#### 2.5.1. Evolution of LDL Concentration

Due to their small size, LDL molecules suffer convection due to plasma flow across the arterial wall. They additionally have diffusion. Once LDL molecules are on the arterial wall, they are oxidized, so their reaction term is:

(13) $$\begin{array}{l} f_{C_{LDL,w}}\left(C_{LDL,w}\right)=-d_{LDL}C_{LDL,w}, \end{array}$$

where *d*_*LDL*_ is the degradation ratio of LDL on the arterial wall, and *C*_*LDL,w*_ its concentration at each time. LDL flow through the endothelium can be calculated with the Kedem-Katchalsky equation (Olgac et al., 2008).

(14) $$\begin{array}{l} J_{S,LDL}=C_{LDL,l}\cdot LDL_{dep}\cdot P_{app}, \end{array}$$

where *C*_*LDL,l*_ is the LDL concentration at the lumen, *LDL*_*dep*_ the quantity of LDL molecules that are deposited into the arterial wall and *P*_*app*_ the coefficient of apparent permeability of the arterial wall, which is composed of the permeability of normal junctions, leaky junctions and vesicular pathways (*P*_*app,nj*_, *P*_*app,lj*_ and *P*_*app,v*_, respectively):

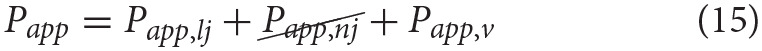



Molecule transport through endothelium occurs in different ways depending on the size of the particles. For molecules with a size lower than 2 nm, transport is allowed through all the possible ways, but for greater molecules (such as LDL, whose size is approximately 11 nm), transport across normal junctions is not allowed, so for this case, molecular transport through the endothelium only occurs by leaky junctions and vesicular pathways.

According to Olgac et al. (2008), molecular transport of LDL through vesicular pathways is 0.1 of the flux through leaky junctions.

(16) $$\begin{array}{l} P_{app,v}=0.1\cdot P_{app,lj} \end{array}$$

The apparent permeability of leaky junctions can be defined as:

(17) $$\begin{array}{l} P_{app,lj}=P_{lj}Z_{lj}+J_{v,lj}\cdot \left(1-\sigma_{f,lj}\right), \end{array}$$

where *P*_*lj*_, *Z*_*lj*_ and σ_*f,lj*_ are, respectively, diffusive permeability of leaky junctions, a factor of reduction of the concentration gradient of LDL at the entrance of flow and the solvent-drag coefficient of leaky junctions. Therefore, LDL flux across the endothelium can be written as:

(18) $$\begin{array}{l} J_{S,LDL}=1.1\cdot C_{LDL,l}\cdot LDL_{dep}\cdot \left(P_{lj}Z_{lj}+J_{v,lj}\left(1-\sigma_{f,lj}\right)\right) \end{array}$$

Diffusive permeability of leaky junctions is defined as:

(19) $$\begin{array}{l} P_{lj}=\frac{A_{p}}{S}\chi P_{slj}, \end{array}$$

where χ is the difference between the total area of endothelial cells and the area of cells separated by leaky junctions, where LDL flux is allowed:

(20) $$\begin{array}{l} \chi =1-\alpha_{lj}, \end{array}$$

where α_*lj*_ is the ratio between the radius of an LDL molecule (*a*_*m*_) and the half-width of a leaky junction (*w*_*l*_):

(21) $$\begin{array}{l} \alpha_{lj}=\frac{a_{m}}{w_{l}} \end{array}$$

Finally, *P*_*slj*_ is the permeability of a leaky junction that can be computed using the equations of Appendix B.

In addition, we have to impose as an additional boundary condition the LDL concentration at adventitia (*C*_*LDL,adv*_) which is obtained from experimental data of Meyer et al. (1996) to comply with the experimental LDL distribution of LDL across the arterial wall.

#### 2.5.2. Evolution of Oxidized LDL Concentration

We considered that once LDL becomes oxidized, it does not experiment convection, but it has the diffusion term. Their reaction terms are due to two factors: the first one refers to LDL that becomes oxidized in the arterial wall, and the second one refers to oxidized LDL that is absorbed by macrophages:

(22) $$\begin{array}{l} f_{C_{LDL_{ox,w}}}\left(C_{LDL,w},C_{LDL_{ox,w}},C_{M,w}\right)=d_{LDL}C_{LDL,w}-LDL_{ox,r}C_{LDL_{ox,w}}C_{M,w}, \end{array}$$

where *C*_*LDL*_*ox,w*__ and *C*_*M,w*_ are oxidized LDL and macrophage concentration at each point of the arterial wall, respectively. *LDL*_*ox,r*_ is the ratio of the quantity of oxidized LDL that a single macrophage absorbs.

#### 2.5.3. Evolution of Monocyte Concentration

Monocytes are cells, so they do not have convection. The first reaction term of the equation corresponds to the monocytes that disappear because of their differentiation into macrophages. The second term is due to apoptosis of monocytes:

(23) $$\begin{array}{l} f_{C_{m,w}}\left(C_{m,w}\right)=-d_{m}C_{m,w}-m_{d}C_{m,w}, \end{array}$$

where *C*_*m,w*_ is monocyte concentration on the arterial wall, *d*_*m*_ a parameter that represents the rate of monocytes that differentiate into macrophages, and *m*_*d*_ monocyte rate of death.

#### 2.5.4. Evolution of Macrophage Concentration

Similar to monocytes, macrophages are cells, so they do not have convection. Their reaction terms are:

(24) $$\begin{array}{l} f_{C_{M,w}}\left(C_{M,w},C_{m,w},C_{LDL_{ox,w}}\right)=d_{m}C_{m,w}-\frac{LDL_{ox,r}}{n_{FC}}\cdot C_{LDL_{ox,w}}C_{M,w}, \end{array}$$

where the first term is the differentiation of monocytes into macrophages and the second one their apoptosis.

*LDL*_*ox,r*_ is the constant rate of oxidized LDL taken up by macrophages, and *n*_*FC*_ is the maximum amount of oxidized LDL that a single macrophage has to ingest to turn into a foam cell. To obtain this value, it was considered that these cells are capable of ingesting particles up to 1.44 times their radius (Cannon and Swanson, 1992), taking into account that the density and molecular weight of LDL are 1,063 $\frac{kg}{m^{3}}$ (Ivanova et al., 2017) and 386.65 $\frac{g}{mol}$ (Guarino et al., 2006), respectively.

#### 2.5.5. Evolution of Cytokine Concentration

Cytokines are proteins, so we did not consider convection for them. In addition, they are surrounded by macrophages, so their diffusion can be considered negligible (Cilla et al., 2014). Cytokine reaction terms are due to their degradation and production:

(25) $$\begin{array}{l} f_{C_{c,w}}\left(C_{c,w},C_{LDL_{ox,w}},C_{M,w}\right)=C_{r}C_{LDL_{ox,w}}C_{M,w}-d_{c}C_{c,w}, \end{array}$$

where *C*_*c,w*_ is cytokine concentration on the arterial wall. *C*_*r*_ is the ratio of cytokine production due to the presence of oxidized LDL and macrophages on the arterial wall, and *d*_*c*_ the cytokine degradation rate.

#### 2.5.6. Evolution of Contractile Smooth Muscle Cell Concentration (CSMC)

Due to the size of CSMCs, they have neither convection nor diffusion. At the beginning of the inflammatory process, all the muscle cells on the arterial wall are of a contractile phenotype, but the presence of cytokines on the arterial wall make them change into a synthetic phenotype, so the reactive term of CSMCs is expressed as follows.

(26) $$\begin{array}{l} f_{C_{CSMC,w}}\left(C_{CSMC,w},C_{c,w}\right)=-C_{CSMC,w}\cdot S_{r}\cdot \left(\frac{C_{c,w}}{k_{c}\cdot C_{c,w}^{th}+C_{c,w}}\right) \end{array}$$

*C*_*CSMC,w*_ is CSMC concentration at the arterial wall. *S*_*r*_ is the CSMC differentiation rate due to the presence of cytokines on the arterial wall, and finally, $C_{c,w}^{th}$ is the maximum cytokine concentration allowed at the arterial wall.

#### 2.5.7. Evolution of Synthetic Smooth Muscle Cell Concentration (SSMC)

Analogous to CSMCs, SSMCs have neither convection nor diffusion. Their reaction terms in the arterial wall are due to differentiation of CSMCs into SSMCs, proliferation and apoptosis of SSMCs:

(27) $$\begin{array}{l} f_{C_{SSMC,w}}\left(C_{SSMC,w},C_{CSMC,w},C_{c,w}\right)=\\ C_{CSMC,w}\cdot S_{r}\cdot \left(\frac{C_{c,w}}{k_{c}\cdot C_{c,w}^{th}+C_{c,w}}\right)+\\ \left(\frac{p_{ss}C_{c,w}}{C_{c,w/2}^{th}+C_{c,w}}\right)C_{SSMC,w}\left(1-\frac{C_{SSMC,w}}{C_{SSMC,w}^{th}}\right)-r_{Apop}\cdot C_{SSMC,w}, \end{array}$$

where *C*_*SSMC,w*_ and $C_{SSMC,w}^{th}$ are SSMC concentration and their maximum allowed concentration at the arterial wall, respectively, *p*_*ss*_ the SSMC proliferation rate and *r*_*Apop*_ the SSMC apoptosis rate.

#### 2.5.8. Evolution of Foam Cell Concentration (FC)

Foam cells have neither convection nor diffusion given that they are large cells. The reaction term is due to apoptosis of macrophages into foam cells and can be written as:

(28) $$\begin{array}{l} f_{C_{FC,w}}\left(C_{LDL_{ox,w}},C_{M,w}\right)=\frac{LDL_{ox,r}}{n_{FC}}\cdot C_{LDL_{ox,w}}C_{M,w} \end{array}$$

All parameters in Equation (28) have already been defined.

#### 2.5.9. Evolution of Collagen Fibres

Finally, collagen fibres are composed of many molecules, so they cannot move between arterial wall pores. Therefore, collagen fibres have neither convection nor diffusion. Reaction terms of collagen fibres are due to collagen segregation due to SSMC presence on the arterial wall and by collagen degradation.

(29) $$\begin{array}{l} f_{C_{G,w}}\left(C_{G,w},C_{SSMC,w}\right)=G_{r}\cdot C_{SSMC,w}-d_{G}\cdot C_{G,w}, \end{array}$$

where *G*_*r*_ and *d*_*G*_ are the collagen secretion and degradation rate, respectively, and *C*_*G,w*_ its concentration on the arterial wall.

### 2.6. Haemodynamical Stimuli to Initiate the Inflammatory Process

Three different mechanical stimuli were analysed in this study as potential triggers for the inflammatory process and predictors to foresee the position and growth of atheroma plaques.

The first one is TAWSS, which is defined as:

(30) $$\begin{array}{l} TAWSS=\frac{1}{T}\int_{o}^{T}|\tau \left(t\right)|\cdot dt, \end{array}$$

where *T* is the period of a cardiac cycle and |τ(*t*)| the magnitude of WSS dependent on time, with WSS defined as:

(31) $$\begin{array}{l} WSS=\sqrt{{\tau_{x}}^{2}+{\tau_{y}}^{2}+{\tau_{z}}^{2}}, \end{array}$$

where τ_*x*_, τ_*y*_ and τ_*z*_ are components of the tangential stress vector appearing in the lumen-wall interface of the model.

**Figure 4:**
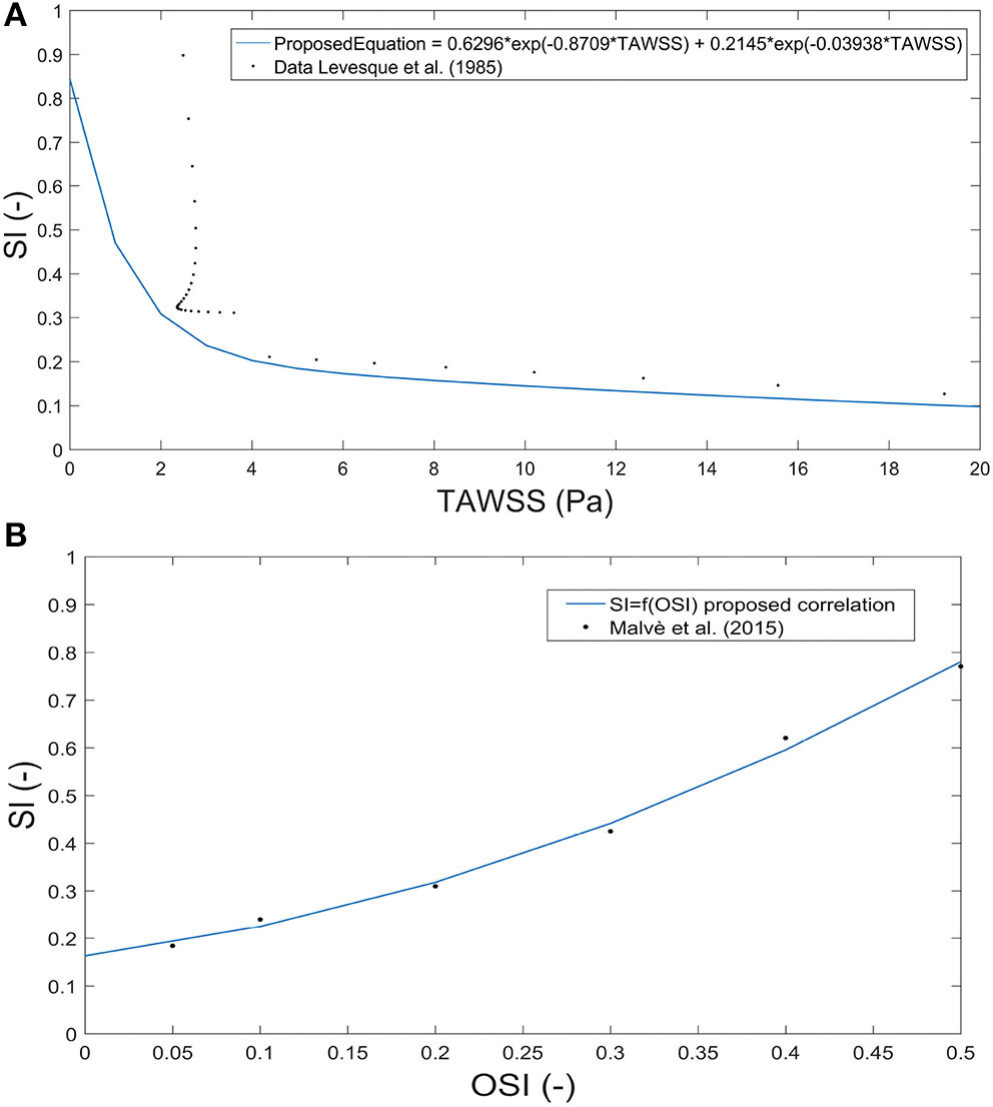
Correlation between the shape index and time average wall shear stress based on experimental data from Levesque et al. (1986) **(A)** and between the shape index and oscillatory shear index based on data from Sáez et al. (2015) **(B)**.

The shape index of endothelial cells directly depends on TAWSS, being proximal to 1 in the case of low TAWSS, meaning that endothelial cells are almost circular. To determine the behaviour of endothelial cells with TAWSS, we propose a numerical correlation based on the experimental results of Levesque et al. (1986). This correlation is shown in Figure 4. The endothelial shape index (SI) is:

(32) $$\begin{array}{l} SI=k_{1}\cdot e^{\left(k_{2}\cdot TAWSS\right)}+k_{3}\cdot e^{\left(k4\cdot TAWSS\right)} \end{array}$$

It is well accepted that areas of low TAWSS are atheroprones. In particular, for carotid arteries, areas below 2 Pa are susceptible to the emergence of atheroma plaques (Zhao et al., 2002; Younis et al., 2004; Filipovic et al., 2013).

The values of the parameters *k*_1_, *k*_2_, *k*_3_, and *k*_4_ are shown in Table 4.

**Table 4:** List of parameters necessary for the correlations of SI as a function of TAWSS, OSI, and the proposed combination of them.

**Adjustment parameters**	
**Parameter**	**Value**
*k*1	0.6296
*k*2	−0.8709
*k*3	0.2145
*k*4	0.03938
*k*5	1.53
*k*6	0.4688
*k*7	0.1631
*k*8	0.0264
*k*9	5.647
*k*10	0.5513
*k*11	−0.1815

The second mechanical stimulus that we considered is OSI:

(33) $$\begin{array}{l} OSI=0.5\cdot \left(1-\frac{\left|\frac{1}{T}\int_{o}^{T}\tau \left(t\right)\cdot dt\right|}{TAWSS}\right) \end{array}$$

SI can be considered directly dependent on OSI; therefore, to determine this behaviour, we propose the next correlation obtained from the experimental data of Levesque et al. (1986). The graphical correlation is shown in Figure 4.

(34) $$\begin{array}{l} SI=k_{5}\cdot OSI^{2}+k_{6}\cdot OSI+k_{7} \end{array}$$

Areas with high OSI are areas in which atheroma plaques are more likely to appear. To estimate the OSI threshold, we first calculated the value of SI corresponding to a value of TAWSS of 2 Pa [Equation (32)], assuming that for this value of SI, the LDL molecules can pass through the endothelium. Therefore, by replacing this SI value in Equation (34), we can estimate that atheroma plaques will grow in areas of OSI higher than 0.1910. The values of the parameters *k*_5_, *k*_6_, and *k*_7_ are shown in Table 4.

**Figure 5:**
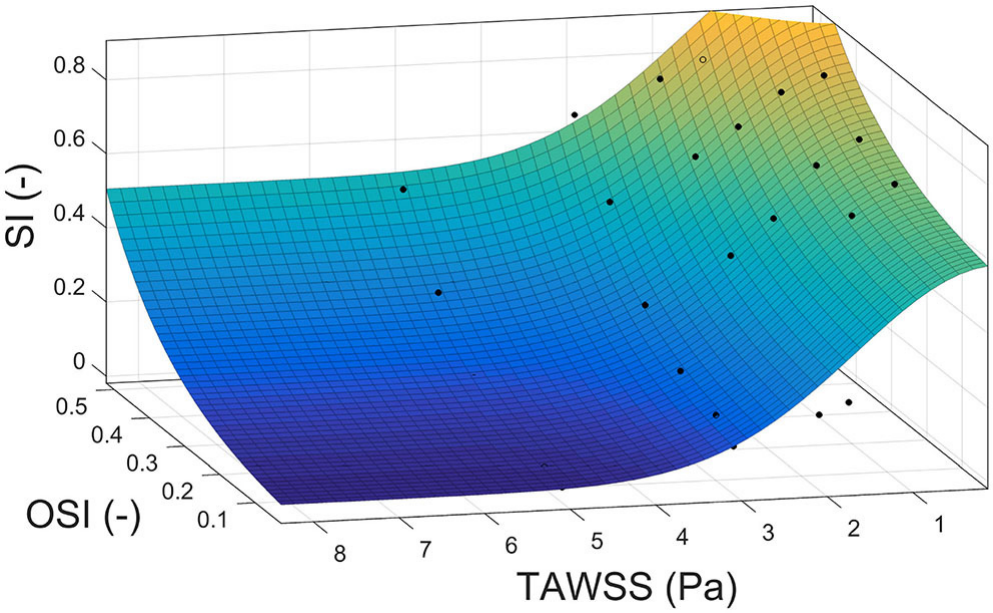
Correlation surface among the shape index, time average wall shear stress and oscillatory shear index based on data from Sáez et al. (2015).

Finally, we proposed a new index to calculate the growth of plaques as a combination of TAWSS and OSI to take into account the effect of both stimuli. For that, we used pseudo-experimental data from Sáez et al. (2015) to approximate the variable SI as a function of TAWSS and OSI, obtaining:

(35) $$\begin{array}{l} SI=k_{8}\cdot e^{k_{9}\cdot OSI}+k_{10}\cdot e^{k_{11}\cdot {\left(TAWSS\right)}^{2},} \end{array}$$

using thresholds obtained before for TAWSS and OSI to determine areas of plaque growth with this new index. The approximation surface is shown in Figure 5. All the adjustment parameters used in the analysis, namely, *k*_8_, *k*_9_, *k*_10_ and *k*_11_, are shown in Table 4.

Monocytes flow from the lumen into the arterial wall and through the endothelium, which also depends on the haemodynamical stimulus that we consider.

For TAWSS, it can be modelled with the Kedem-Katchalsky equation as (Malek and Alper, 1999; Gijsen et al., 2008):

(36) $$\begin{array}{l} J_{s,m}\left(TAWSS\right)=\frac{m_{r}}{\left(1+\frac{TAWSS}{TAWSS_{0}}\right)}\cdot C_{LDL,ox,w}C_{m,l}, \end{array}$$

where *m*_*r*_ is monocyte recruitment from the lumen to the endothelium. TAWSS was modelled as a sigmoid function with maximal and minimal values equal to 2 and 0 Pa, respectively, to allow LDL flux across the endothelium. To completely define the sigmoid, an average value called *TAWSS*_0_ of 1 Pa is necessary.

For the case of using OSI as the mechanical stimulus, another equation was developed in terms of the maximal and minimal fluxes of monocytes obtained with the TAWSS equation:

(37) $$\begin{array}{l} J_{s,m}\left(OSI\right)=m_{r}\cdot \left(8.503\cdot OSI^{2}-3.741\cdot OSI+0.7449\right)\cdot C_{LDL,ox,w}C_{m,l} \end{array}$$

The same procedure was done for the combination of TAWSS and OSI:

(38) $$\begin{array}{l} J_{s,m}\left(TAWSS,OSI\right)=m_{r}\cdot (0.8588\cdot e^{-0.6301\cdot TAWSS}\\ \;+0.1295\cdot e^{3.963\cdot OSI})\cdot C_{LDL,ox,w}C_{m,l} \end{array}$$

### 2.7. Plaque Initiation and Growth

The mass balance for open systems can be written as:

(39) $$\begin{array}{l} \frac{\partial \rho_{o}^{i}}{\partial t}=\Pi_{i}-\nabla \cdot M_{i}, \end{array}$$

where ρ_*o*_ is the total density of the tissue in the reference configuration (Garikipati et al., 2004), Π_*i*_ are the source/sinks and *M*_*i*_ the mass fluxes of the *i* arbitrary species. Π_*i*_ are related to migration, proliferation, differentiation and apoptosis of the cells and secretion and degradation of the substances. The concentrations of each species have the property $\rho_{o}^{i}$, where $\rho_{o}=\sum_{i}\rho_{o}^{i}$ is the total material density of the tissue as the sum over all *i*. The densities, $\rho_{o}^{i}$, change as a result of mass transport and inter-conversion of species, implying that the total density in the reference configuration, ρ_*o*_, changes with time.

As mass transport alters the reference density, $\rho_{o}^{i}$, assuming that these volume changes are isotropic, it leads to the following growth kinematics ${\dot{\text{F}}}_{g}^{i}=\frac{{\dot{\rho }}_{o}^{i}}{\rho_{orig}^{i}}\text{I}$ where $\rho_{orig}^{i}$ means the original concentration of a specie in the undeformed configuration (Garikipati et al., 2004) and **I** is the second-order unit tensor. For a small strain hypothesis and isotropic growth, we can write:

(40) $$\begin{array}{l} \nabla \cdot v^{i}=\frac{{\dot{\rho }}_{o}^{i}}{\rho_{orig}^{i}}, \end{array}$$

where *v* is the velocity of the material points.

Finally, knowing all substance distributions in the arterial wall, we can compute the growth of plaques. The arterial wall change of volume is due to the contribution of all the cells and substances that are present in the inflammatory process, but the influence of most of them is negligible, so we considered that only larger cells and collagen contribute to plaque formation. Therefore, only FCs, SSMCs and collagen fibres contribute to plaque volume in our model.

In addition, we considered isotropic growth of plaques, so atheroma plaque volume change can be written as:

(41) $$\begin{array}{l} \nabla \cdot v=\frac{\partial C_{FC,w}}{\partial t}\cdot Vol_{FC}+\frac{\partial \Delta C_{SSMC,w}}{\partial t}\cdot Vol_{SSMC}+\frac{\partial C_{G,w}}{\partial t}\cdot \frac{1}{\rho_{G}}, \end{array}$$

where ∂*C*_*i,w*_ is the concentration variation with respect to the initial concentration of the considered substance. *Vol*_*FC*_ and *Vol*_*SSMC*_ are volumes of an FC and an SSMC, respectively, which can be calculated by knowing their radius. Finally, ρ_*G*_ is collagen density.

Foam cells were assumed to have a spherical geometry, whereas synthetic smooth muscle cells were modelled as ellipsoids, so their volumes can be calculated with Equations (42), (43).

(42) $$\begin{array}{l} Vol_{FC}=\frac{4}{3}\pi {R_{FC}}^{3} \end{array}$$

(43) $$\begin{array}{l} Vol_{SSMC}=\frac{4}{3}\pi {R_{SSMC}}^{2}\cdot l_{SSMC}, \end{array}$$

where *R*_*FC*_ and *R*_*SSMC*_ are the FC and SSMC radii, respectively, and *l*_*SSMC*_ its length. The parameters for the growth of plaques process are shown in Table 5.

**Table 5:** List of parameters necessary to compute plaque growth in the carotids.

**Plaque growth parameters**			
**Parameter**	**Description**	**Value**	**Reference**
*R*_*FC*_	Foam cell radius	15.264 μ*m*	Krombach et al., 1997
*R*_*SSMC*_	SSMC radius	3.75 μ*m*	Martini, 2012
*l*_*SSMC*_	SSMC length	115 μ*m*	Martini, 2012
ρ_*G*_	Collagen density	$1,000\frac{kg}{m^{3}}$	Sáez et al., 2013

To validate the results, the stenosis ratio (SR) in the areas with maximum plaque was computed, defining the stenosis ratio as the percent area stenosis in a section. It relates the area of the healthy lumen without the presence of plaque with the area of the lumen with plaque, and can be calculated as:

(44) $$\begin{array}{l} SR\left(\%\right)=\left(1-\frac{\text{Lumen area with plaque}}{\text{Lumen area without plaque}}\right)\cdot 100 \end{array}$$

As can be seen, although the geometries are patient-specific, the parameters are based in literature due to the impossibility of determine their value for each patient. Therefore, there is some variability in them, which was checked to see how it can affect to the model. The parameters that are related to LDL have more influence in the plaque growth given that LDL is the substance that initiates all the process. These parameters were calibrated with the patient “A” and used later for the rest of geometries. In other cases, such as the parameters referred to the cell size, an average value of the parameters given in literature was taken.

Regarding measurable parameters for each patient, the most important parameters whose variation would suppose a different behaviour of the model are LDL and monocytes concentration in blood, as well as the arterial pressure of the patient since there are studies that correlate changes in the endothelial permeability as a function of the arterial pressure (Tedgui and Lever, 1984), and other factors, e.g., if the patients are taking medication or not.

In the case of different vascular regions with the same arterial pressure, the parameters that could vary are the hydraulic conductivity of the normal junctions, Lp, nj (Tedgui and Lever, 1984), the monocytes recruitment, mr (Steinberg et al., 1997), as well as the thickness of the arterial wall (Olgac et al., 2008; Sommer et al., 2010).

## 3. Results

In Figure 1, we can see the seven real geometries with their corresponding plaques indicated by arrows and the healthy one without plaque. All the geometries, with the exception of E, have large atheroma plaques at both the CCA close to the bifurcation and at the ICA. Geometry E presents a unique plaque in the ICA.

**Figure 6:**
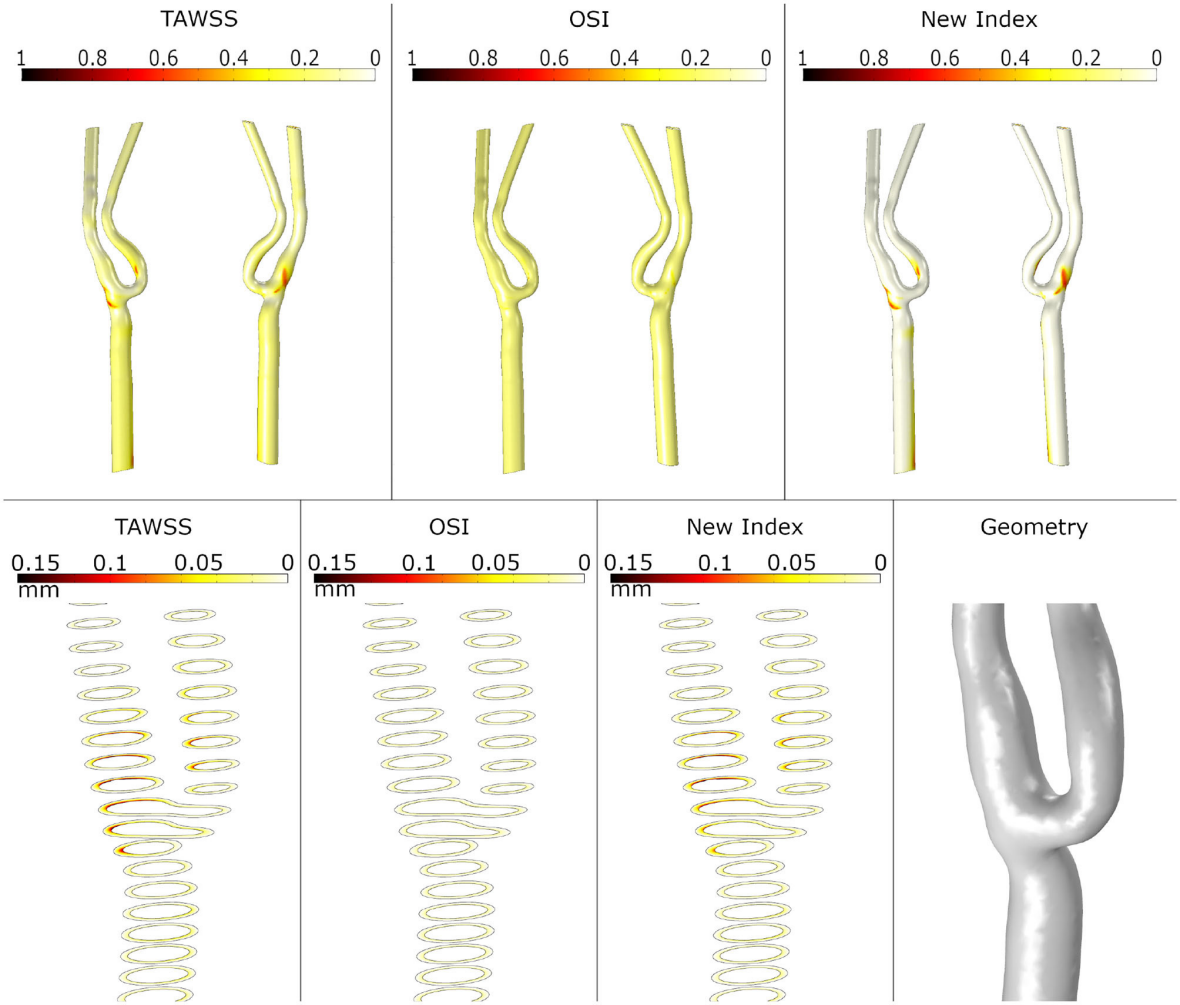
Shape index distribution (first row) and detail of the growth of plaque in the bifurcation area (second row) in the healthy geometry with the different mechanical stimuli studied. The first column depicts the use of TAWSS, the second column the OSI and the last column the proposed combination of TAWSS and OSI.

First, we analysed the haemodynamical stimuli effect and the growth on the healthy artery. The results of the simulation for SI and growth of plaques computed with TAWSS, OSI and the new proposed variable are presented in Figure 6. As we expected, small areas with high SI are usually accepted as atheroprones - presented in the healthy geometry - and the corresponding growth is reduced. In particular, for OSI stimulus, negligible growth is presented.

**Figure 7:**
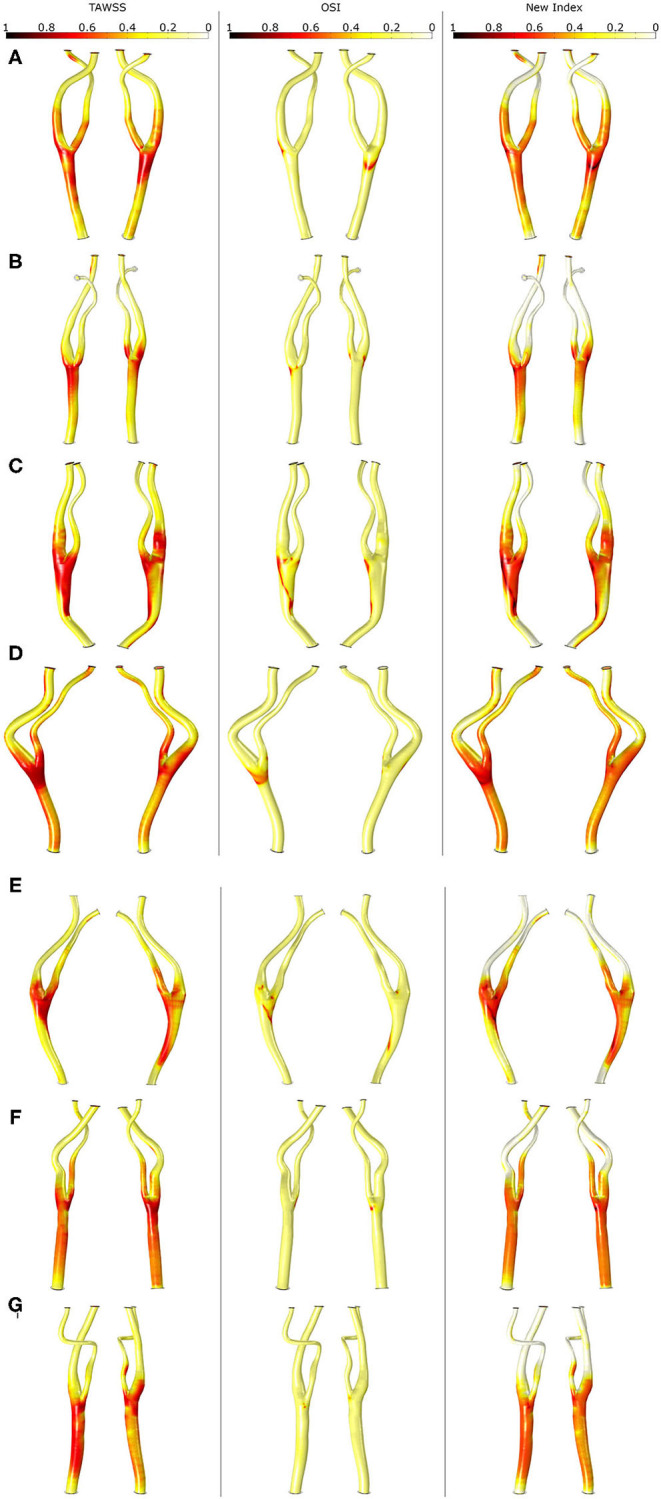
Shape index distribution in all the geometries **(A–G)** with the different mechanical stimuli studied. The first column depicts the use of TAWSS, the second column the OSI and last column the proposed combination of TAWSS and OSI.

The SI obtained with TAWSS, OSI and the new variable is represented for all the pathological carotid bifurcations in Figure 7. As seen, OSI predicts these areas with high values near the bifurcation but lower than TAWSS, which also predicts areas of high SI in the CCA close to the bifurcation as well as in some areas of ICA and ECA. Finally, the new index also predicts high SI in these areas but in a more localized way than TAWSS. Note that for E geometry, none of the stimuli predicts the location of the plaque.

**Figure 8:**
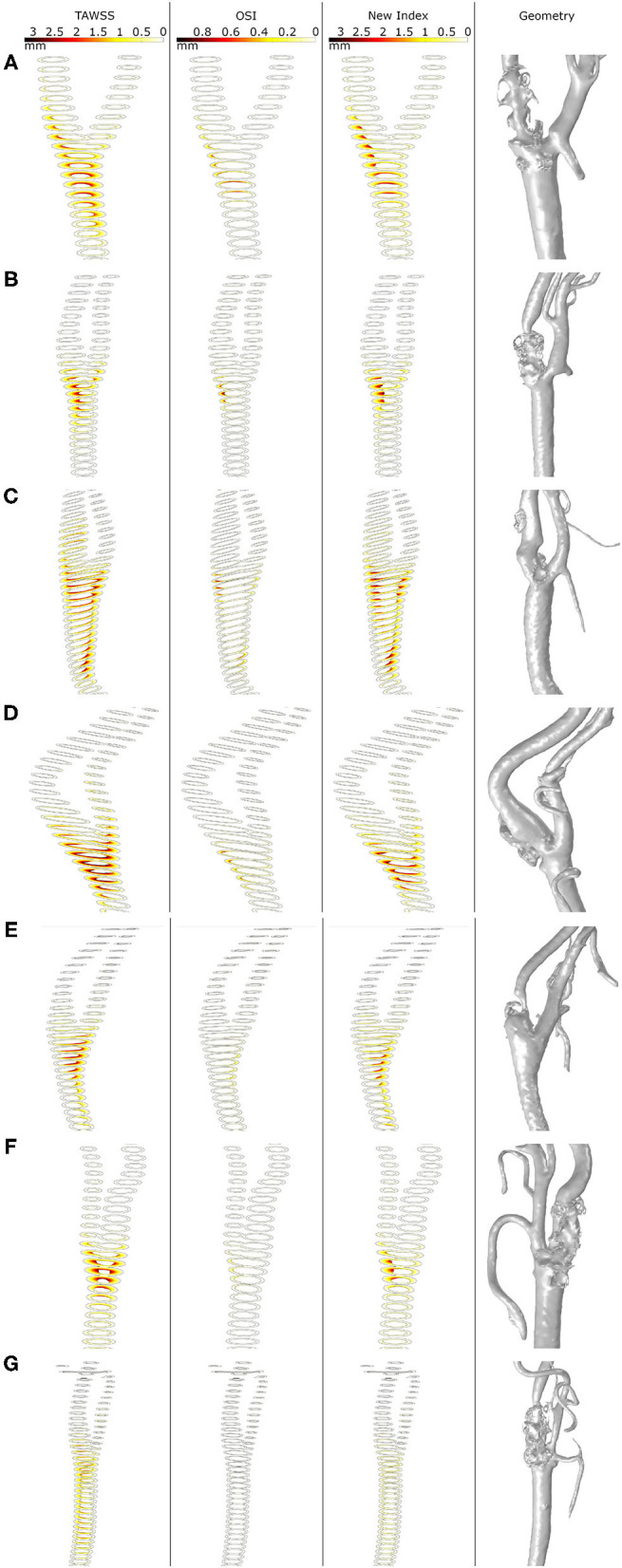
Growth of plaques in all the geometries **(A–G)** with the different mechanical stimuli studied. The first column depicts the use of TAWSS, the second column the OSI, the third column the proposed combination of TAWSS and OSI and the last column the real plaques of the patients.

In Figure 8, growth of plaques after 30 years of the inflammatory process is represented for all the geometries considering the different haemodynamical stimuli. Generally, the location of plaques was better estimated using the new proposed stimulus. For all cases, OSI underestimates the area of the plaques and shows the worst prediction of the location. TAWSS predicts non-physiological growth on the CCA due to the very low values of WSS in this area. For patients A, C and D, the location of the plaque matches the clinical evidence using only the new stimulus and the prediction fails for TAWSS and OSI. For patient B, the new index predicts the location of the plaque of the ICA but fails on the length of the disease. For patient C, the new stimulus matches the location of the plaques on ICA and ECA; however, it predicts plaque on CCA that it is not presented on the clinical images, likely due to an excessive influence of TAWSS in this area. Plaques on the ICA where not predicted by any of the stimuli for patients E and G.

Finally, Table 6 shows the computational stenosis ratio obtained using our mechanobiological model for all the carotid bifurcations and compared with the real ones taken from the clinical images. It can be observed that the stenosis ratio after 30 years was better predicted using the new variable than the other two for the seven studied patients. For example, the stenosis ratio predicted with the new variable for patient C were 52.65 and 22.71 % for the CCA and ICA, respectively, and the real values are 61.14 and 32.91 % for the CCA and ICA, respectively, showing differences of 8.49 and 10.2 % of stenosis, respectively. In general, for all geometries, the new variable has values of maximal and minimal errors of estimated stenosis of 32.89 and 2.77 %, respectively. TAWSS overestimates the stenosis ratio with a maximal and minimal error of 45.74 and 3.58 %, respectively. In contrast, OSI underpredicts the stenosis ratio with an error higher than 9.53 % for all cases.

**Table 6:** Stenosis ratio computed for all the geometries with all the mechanical stimuli and the corresponding ratio on the clinical images.

**Stenosis ratio (%)**					
	**Location**	**TAWSS**	**OSI**	**New variable**	**Real**
Geom. A	CCA	59.57	6.49	39.35	13.82
	ICA	30.50	2.82	25.30	70.45
Geom. B	CCA	47.42	2.98	36.12	18.41
	ICA	28.15	1.93	20.22	39.56
Geom. C	CCA	47.10	10.30	52.65	61.14
	ICA	36.49	1.33	22.71	32.91
Geom. D	CCA	63.03	8.61	47.05	52.39
Geom. E	CCA	23.61	1.81	15.84	19.61
Geom. F	CCA	63.55	2.44	36.28	33.51
Geom. G	CCA	42.14	0.02	21.45	9.55
	ICA	26.44	2.33	12.91	45.81

## 4. Discussion

This work extends the mechanobiological model developed by Cilla et al. (2014) from an axisymmetric to a 3D model and is essential as a prior step to its application to patient-specific images. It is worth highlighting that atheroma plaques are usually eccentric, and this feature cannot be captured with 2D axisymmetric models. This works also updates some terms of the equations to enhance its convergence (apoptosis of macrophages into foam cells, differentiation of CSMCs into SSMCs and proliferation of SSMCs, and adding a new term in SSMCs due to their apoptosis) and is solved in 3D instead an axisymmetric formulation. However, the computational cost is higher, and some simplifications are necessary. Another improvement of this model is the computation of blood flow in the transient mode.

This transient mode for the blood flow allows us to analyse and compare some of the most common mechanical stimuli that are normally used for predicting atheroma plaque locations, TAWSS and OSI. We also propose a new mechanical stimulus as a combination of TAWSS and OSI to better predict the location of plaques. The model was used to check which mechanical stimulus is more appropriate to predict the location of atheroma plaques on carotid geometries.

The model was computed in a healthy geometry without significant plaque in which there is no relevant haemodynamical stimuli to develop atheroma plaques to verify that the model is stable and plaques only grow when there is an atheroprone stimulus. Additionally, atheroma plaque growth was computed in different geometries obtained from pathological patient-specific images in order to obtain the equivalent healthy geometries to validate the results of the model.

As we can see in Figure 8, plaques predicted with different haemodynamical stimulus growth in different locations and present distinct stenosis ratio; thus, the choice of the haemodynamical stimulus to predict the location of plaque results crucial. TAWSS predicts excessively large plaques in the CCA branch of the carotids, far from the bifurcation, that do not match with the real ones. On the other hand, TAWSS adequately predicts the size of plaques appearing in its own bifurcation location compared with the real geometries. In contrast, OSI locates plaques with a better precision than TAWSS, but the growth ratio is very low in comparison to the actual ones. Finally, the new variable proposed in this study combines the results achieved with TAWSS and OSI, predicting in a more adequate way the location of atheroma plaques as in OSI - limiting the growth in areas of the CCA far from the bifurcation- and its growth is similar to the real one observed in the clinical images, similar to TAWSS.

The growth model fails to predict the location and size when haemodynamics cannot predict high SI on the location of the plaques. Although most of plaques predicted with the model correspond to the ones clinically observed, there are a few plaques that cannot be explained with this model. For patients E and G, the stenosis appears in a zone where no haemodynamical disturbance is observed; therefore in these patients, haemodynamics are not the main trigger of the disease and could be due to other systemic or genetic conditions of the patient in this location, e.g., external lesion or pathological weakness of the intimal layer.

**Figure 9:**
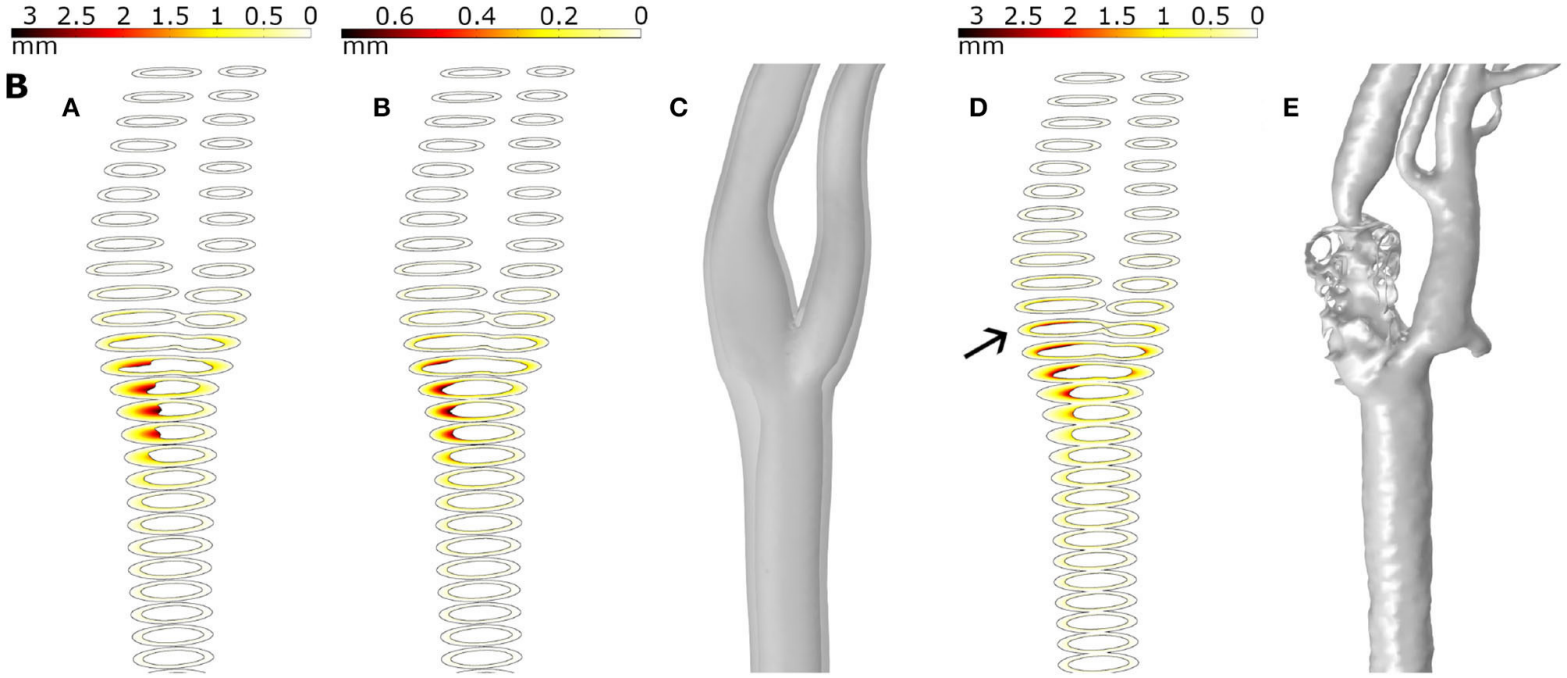
Results for plaque growth in patient B with 30 and 15 years of continuous process (**A,B**, respectively), the updated geometry after 15 years of the growth process **(C)**, the growth after 30 years in the updated geometry **(D)** and the real plaque of the patient **(E)**.

Even though the model correctly predicts the plaques appearing in the specific bifurcation and the ECA, some plaques presented at the ICA are not very well predicted by any mechanical stimulus studied in this work. We hypothesized that it could be due to the uncoupled form between haemodynamics and growth, where fluid is computed at the beginning of the process and it is not updated with the plaque growth. First, plaques appear at the CCA branch, and then they cause a change of the blood flow downstream and the variable stimuli -TAWSS and OSI- could be affected at an area behind them and a new plaque is likely to appear in the ICA. To validate this hypothesis, we computed the growth of B geometry until 15 years, and then we actualized the geometry and computed it again. In Figure 9, we can see the different images that represent growth for a total of 30 years, growth for only half of the time (15 years), reconstruction of an updated geometry of the bifurcation including the stenosis for 15 years, growth for 30 years with the updated geometry and finally the actual clinical image. It can be observed that the plaque in the ICA better matches the real plaque. Due to the high computational cost of the 3D version of our mechanobiological model, full FSI for 3D real carotid images is not available.

As seen, the location of plaques was better predicted than the stenosis ratio, which was predicted with errors between 2.77 and 32.89 %, depending on the analysed geometry. This could be because the location of plaques only depends on haemodynamics, while the stenosis ratio also depends on specific parameters of the patients that are unknown, such as real concentration of LDL in the blood and the time that they have had high LDL levels. Moreover, it is also dependent on specific parameters of the inflammatory model. It could obviously rely on other factors not taken into account in the model that could affect plaque growth, e.g., age, gender, blood pressure level or genetic conditions. However, in general, our model using the new haemodynamic stimulus should predict the location and the growth of the plaques.

Apart from the LDL molecules, many studies have focused on the governing mechanics interaction of the different biological species that play a role in the atheroma plaque development from a computational (see e.g., Ougrinovskaia et al., 2010; Di Tomaso et al., 2011, among others) point of view. Furthermore, there are greatly varying degrees of complexity in these computational studies depending on the number of species considered and the development of the equations proposed. (Zohdi et al., 2004) modelled the adhesion of monocytes to the endothelial surface, which is controlled by the intensity of the blood flow and the adhesion molecules stimulated by the excess of LDL, the penetration of the monocytes into the intima and subsequent inflammation of the tissue, and the rupture of the plaque accompanied by some degree of thrombus formation or even subsequent occlusive thrombosis. Their modelling approach predicts a priori the time to rupture as a function of arterial geometry, diameter of the monocyte, adhesion stress, bulk modulus of the ruptured wall material, blood viscosity, flow rate and mass density of the monocytes. Di Tomaso et al. (2011) considered the interaction between just two species, LDL and monocytes, but the monocyte behaviour was modelled in a very simple way. Fok (2012) proposed a mathematical model of intimal thickening, posed as a free boundary problem. Intimal thickening was driven by damage to the endothelium, resulting in the release of cytokines and migration of SMCs. More complex studies were presented by Siogkas et al. (2011), who included in their model oxidized LDL, macrophages and cytokines, considering that all the LDL molecules and the monocytes were oxidized and differentiated, respectively, at the instant in which these agents pass through the endothelium. A similar study was presented by Calvez et al. (2009) from a mathematical point of view, but their study also included the foam cell. Ougrinovskaia et al. (2010) explored the uptake of cholesterol by different scavenger receptors of macrophages during early-stage atherosclerosis using an ordinary differential equation (ODE) model. It was found that macrophage proliferation rather than an increased influx of LDL particles drives lesion instability. Finally, Bulelzai and Dubbeldam (2012) presented a qualitative mathematical model consisting of a number of ordinary differential equations for the concentrations of the most relevant constituents of the atherosclerotic plaque: macrophages, monocytes, foam cell and oxidized LDL. More complex 2D mechanobiological models have been presented by other authors. For example, Filipovic et al. (2011) used axisymmetric models and thus they could not obtain eccentric plaques. In other studies, such as Filipovic et al. (2013), they used three-dimensional carotid geometries with Kedem-Katchalsky equations and convection-diffusion-reaction equations, but they only considered three substances on the arterial wall and focused the study in the location of plaques and not in their growth. Alimohammadi et al. (2017) and Díaz-Zuccarini et al. (2014) used the aortic bifurcation and the left femoral artery, respectively, in their studies and focused more on the location of plaques than on their growth. Only Alimohammadi et al. (2017) analysed the typical haemodynamical stimuli TAWSS or OSI and proposed a combined index, termed HOLMES, to emphasize regions of highly oscillatory and low-magnitude WSS; however, they focused only on the location of calcifications.

Moreover, it is important to highlight the advantages and disadvantages of our model versus agent-based models (Bhui and Hayenga, 2017; Corti et al., 2020). The main advantage of our model against versus agent-based models is that using a continuum model allows us to simulate the plaque growth in a real complex 3D geometry with an accessible computational cost. Moreover, average values of the parameters and sensitivity analysis are easily implemented. However, the disadvantage is that with the continuum models we cannot take into account the random behaviour of the cells and relation with micro-constituents, which is the one of the main strengths of agent-based models.

The findings of this study should be interpreted within the context of its limitations. For example, our model would be improved by implementing a fluid-structure interaction to better approximate the real pathology. Concerning the mathematical model, only the main processes were included in the model, while other important processes in the development of the atheroma plaque, such as the degradation of collagen with age, were considered. The haemodynamics were considered the main trigger of atherosclerosis initiation. Thus, the cyclic stretch effects of vessel compliance or curvature were disregarded. Another limitation of our model is that we did not have the geometries before plaques were developed, so the real geometries without plaques were unknown. It is a limitation because the real healthy geometries can have some differences with the reconstructed ones and it can have an influence in the obtained results. We did not have real plaque growth monitoring to see plaque geometry evolution, and, in the same way, we also did not have real data from each patient, e.g., blood pressure and flow, LDL levels and number of years with high LDL levels and other pathologies that they may have, so we did not use patient-specific parameters to solve the problem. A fixed plaque growth of 30 years was considered for our analysis based in literature (Insull, 2009). In addition, there are no experimental data about OSI and TAWSS and OSI combination influences on SI, and we used a simplified model in which we only consider the more important substances for plaque growth and do not consider different kinds of cytokines (IL-4, IL-10, IL-13, or TFG beta) or T-cells or free radicals that oxidize LDL. Adjusted parameters come from different species and vessels in which the studies were developed, and they were not all human carotids. We also did not consider other processes that may have an important role in atherosclerosis, such as mechanotaxis. This model assumes that the substances can move from the lumen to the arterial wall, but not in the reverse direction, and the transport properties are set as constant, but in fact, they are very likely to change during plaque formation.

In conclusion, despite the limitations, our model can predict the location and the growth of plaques in the main cases. The results show that prediction of plaques is, in most cases, better using the new mechanical stimulus proposed in this study than using TAWSS or OSI, with a maximal error of 32.89 % on the stenosis ratio computed at the areas of higher occlusion of the lumen due to the plaques. Based on the results, it can be concluded that the functional regulation of the endothelium by local haemodynamic shear stress provides a model for understanding the focal propensity of atherosclerosis in the setting of systemic factors and this may help to guide future therapeutic strategies.

In the future, the model could be used to predict if a patient is susceptible to develop atheroma plaques and if so, to determine the places where plaques are likely to appear. In this way, it would be possible to take the necessary treatment to prevent atheroma plaque development and all the consequences derived from atherosclerosis.

## Data Availability Statement

The raw data supporting the conclusions of this article will be made available by the authors, without undue reservation.

## Author Contributions

MC, MM, and EP conceived and designed the study. PH-L, MC, MM, and EP development of the mathematical model. PH-L computational implementation of the model and postprocesing the results. PH-L, MC, MM, and EP writing, review, and editing.

## Conflict of Interest

The authors declare that the research was conducted in the absence of any commercial or financial relationships that could be construed as a potential conflict of interest.

## Appendix A

Hydraulic conductivity of leaky junctions can be calculated with the equation (A1):

(A1) $$\begin{array}{l} Lp_{lj}=\frac{Ap}{S}\cdot Lp_{slj}, \end{array}$$

where $\frac{Ap}{S}$ is the fraction of total area occupied by leaky junctions and *Lp*_*slj*_ is the hydraulic conductivity of a single leaky junction. To determine the value of these parameters, we considered that the spaces between endothelial cells have a cylindrical shape, while leaky junctions have a ring shape and they are evolving the leaky cells (Weinbaum et al., 1985; Yuan et al., 1991). We assumed that leaky junctions are aleatory distributed with a distance between them of 2ϵ_*lj*_, where 2ϵ_*lj*_ is the permeability of a leaky junction (Weinbaum et al., 1985; Yuan et al., 1991). Therefore, the circumferences of radius ϵ_*lj*_ can be traced periodically with a leaky cell in their centre.

According to this, the fraction of area occupied by leaky junctions can be defined as:

(A2) $$\begin{array}{l} \frac{A_{p}}{S}=\frac{A_{slj}}{\pi \cdot {\epsilon_{lj}}^{2}}, \end{array}$$

where $\pi \cdot {\epsilon_{lj}}^{2}$ is the total area that corresponds to leaky junctions (because they are separated by a distance of 2ϵ_*lj*_) and *A*_*slj*_ is the area of a single leaky junction that can be calculated with equation (A3), assuming a simplification for reduced thickness:

(A3) $$\begin{array}{l} A_{slj}=2\pi \cdot R_{cell}\cdot 2w_{l}, \end{array}$$

where *R*_*cell*_ is the radius of endothelial cells, and *w*_*l*_ the half-width of a leaky junction (Weinbaum et al., 1985; Yuan et al., 1991; Huang et al., 1994).

On the other hand, the ratio of the area occupied by leaky cells and the total of cells, Φ_*lj*_, can be defined as (Weinbaum et al., 1985; Huang et al., 1994):

(A4) $$\begin{array}{l} \Phi_{lj}=\frac{{R_{cell}}^{2}}{{\epsilon_{lj}}^{2}} \end{array}$$

Therefore, combining the equations (A2) (A3) and (A4), we can obtain the expression to calculate the area occupied by leaky junctions (A5):

(A5) $$\begin{array}{l} \frac{A_{p}}{S}=\frac{4w_{l}}{R_{cell}}\cdot \Phi_{lj} \end{array}$$

Nevertheless, the ratio Φ_*lj*_ is not known because ϵ_*lj*_ is also not known, but it is known that it is a function of the considered mechanical stimulus, which can be calculated by knowing different tangential stresses in the arterial wall caused by blood flow during a cardiac cycle.

## Appendix B

*P*_*slj*_ is the permeability of a leaky junction, which can be calculated with the diffusion coefficient of LDL in a leaky junction (*D*_*lj*_) and the length of a leaky junction (*l*_*lj*_):

(A6) $$\begin{array}{l} P_{slj}=\frac{D_{lj}}{l_{lj}} \end{array}$$

The diffusion coefficient of a leaky junction is related to the LDL diffusion coefficient with the following empirical correlation (Olgac et al., 2008):

(A7) $$\begin{array}{l} \frac{D_{lj}}{D_{l}}=F\left(\alpha_{lj}\right)=1-1.004\alpha_{lj}+0.418\alpha_{lj}^{3}-0.16\alpha_{lj}^{5} \end{array}$$

The reduction factor of the concentration gradient of LDL depends on a modified Peclet number:

(A8) $$\begin{array}{l} Z_{lj}=\frac{Pe_{lj}}{e^{\left(Pe_{lj}\right)}-1}, \end{array}$$

which is defined as:

(A9) $$\begin{array}{l} Pe_{lj}=\frac{Jv,lj\cdot \left(1-\sigma_{f,lj}\right)}{P_{lj}} \end{array}$$

Finally, the solvent-drag coefficient of leaky junctions is given by (Olgac et al., 2008) V

(A10) $$\begin{array}{l} \sigma_{f,lj}=1-\frac{2}{3}\alpha_{lj}^{2}\left(1-\alpha_{lj}\right)\cdot F\left(\alpha_{lj}\right)-\left(1-\alpha_{lj}\right)\left(\frac{2}{3}+\frac{2\alpha_{lj}}{3}-\frac{7\alpha_{lj}^{2}}{12}\right) \end{array}$$

## References

[B1] Ai L. Vafai K. (2006). A coupling model for macromolecule transport in a stenosed arterial wall. Int. J. Heat Mass Trans. 49, 1568–1591. 10.1016/j.ijheatmasstransfer.2005.10.041

[B2] Alimohammadi M. Pichardo-Almarza C. Agu O. Díaz-Zuccarini V. (2017). A multiscale modelling approach to understand atherosclerosis formation: a patient-specific case study in the aortic bifurcation. Proc. Inst. Mech. Eng. H J. Eng. Med. 231, 378–390. 10.1177/0954411917697356 28427316 PMC5405845

[B3] Arshad M. Ghim M. Mohamied Y. Sherwin S. J. Weinberg P. D. (2020). Endothelial cells do not align with the mean wall shear stress vector. J. R. Soc. Interf. 18:20200772. 10.1098/rsif.2020.0772 33435845 PMC7879765

[B4] Bennett M. R. Evan G. . Schwartz S. M. (1995). Apoptosis of human vascular smooth muscle cells derived from normal vessels and coronary atherosclerotic plaques 2274.) key words: apoptosis * atherosclerosis * vascular smooth muscle * bcl-2. Clin. Invest 95, 2266–2274. 10.1172/JCI117917 7738191 PMC295839

[B5] Bhui R. Hayenga H. N. (2017). An agent-based model of leukocyte transendothelial migration during atherogenesis. PLoS Comput. Biol. 13, 1–23. 10.1371/journal.pcbi.1005523 28542193 PMC5444619

[B6] Boyle C. J. Lennon A. B. Prendergast P. J. (2011). In silico prediction of the mechanobiological response of arterial tissue: application to angioplasty and stenting. J. Biomech. Eng. 133, 1–10. 10.1115/1.4004492 21950894

[B7] Budu-Grajdeanu P. Schugart R. C. Friedman A. Valentine C. Agarwal A. K. Rovin B. H. (2008). A mathematical model of venous neointimal hyperplasia formation. Theor. Biol. Med. Model. 5, 1–9. 10.1186/1742-4682-5-2 18215280 PMC2263040

[B8] Bulelzai M. A. K. Dubbeldam J. L. A. (2012). Long time evolution of atherosclerotic plaques. J. Theor. Biol. 297, 1–10. 10.1016/j.jtbi.2011.11.023 22142625

[B9] Calvez V. Ebde A. Meunier N. Raoult A. (2009). Mathematical modelling of the atherosclerotic plaque formation. ESAIM Proceedings 28, 1–12. 10.1051/proc/2009036

[B10] Cannon G. J. Swanson J. A. (1992). The macrophage capacity for phagocytosis. J. Cell Sci. 101, 907–913. 10.1242/jcs.101.4.907 1527185

[B11] Caro C. G. Pedley T. J. Schroter R. C. Seed W. A. (1978). The Mechanics of the Circulation. Oxford, UK: Oxford University Press.

[B12] Chamley-Campbell J. H. Campbell G. R. Ross R. (1981). Phenotype-dependent response of cultured aortic smooth muscle to serum mitogens. J. Cell Biol. 89, 378–383. 10.1083/jcb.89.2.379 7251658 PMC2111686

[B13] Chien S. (2003). Molecular and mechanical bases of focal lipid accumulation in arterial wall. Progr. Biophys. Mol. Biol. 83, 131–151. 10.1016/S0079-6107(03)00053-1 12865076

[B14] Cilla M. Peña E. Martínez M. A. (2014). Mathematical modelling of atheroma plaque formation and development in coronary arteries. J. R. Soc. Interface 11:20130866. 10.1098/rsif.2013.0866 24196695 PMC3836327

[B15] Corti A. Chiastra C. Colombo M. Garbey M. Migliavaca F. Casarin S. (2020). A fully coupled computational fluid dynamics - agent-based model of atherosclerotic plaque development: multiscale modeling framework and parameter sensitivity analysis. Comput. Biol. Med. 118:103623. 10.1016/j.compbiomed.2020.103623 31999550

[B16] Dabagh M. Jalali P. Konttinen Y. T. (2009). The study of wall deformation and flow distribution with transmural pressure by three-dimensional model of thoracic aorta wall. Med. Eng. Phys. 31, 816–824. 10.1016/j.medengphy.2009.03.005 19356969

[B17] Dai G. H. Kaazempur-Mofrad M. R. Natarajan S. Zhang Y. Z. Vaughn S. Blackman B. R.. (2004). Distinct endothelial phenotypes evoked by arterial waveforms derived from atherosclerosis-susceptible and -resistant regions of human vasculature. Proc. Natl. Acad. Sci. U.S.A 101, 14871–14876. 10.1073/pnas.0406073101 15466704 PMC522013

[B18] Di Tomaso G. Diaz-Zuccarini V. Pichardo-Almarza C. (2011). A multiscale model of atherosclerotic plaque formation at its early stage. IEEE Trans. Biomed. Eng. 58, 3460–3463. 10.1109/TBME.2011.2165066 21859610

[B19] Díaz-Zuccarini V. Di Tomaso G. Agu O. Pichardo-Almarza C. (2014). Towards personalised management of atherosclerosis via computational models in vascular clinics: Technology based on patient-specific simulation approach. Health. Technol. Lett. 1, 13–18. 10.1049/htl.2013.0040 26609369 PMC4613843

[B20] Filipovic N. Rosic M. Tanaskovic I. Parodi O. Fotiadis D. (2011). in the Arteries. 1986, 195–198. 10.1109/IEMBS.2011.6090031 22254283

[B21] Filipovic N. Teng Z. Radovic M. Saveljic I. Fotiadis D. Parodi O. (2013). Computer simulation of three-dimensional plaque formation and progression in the carotid artery. Med. Biol. Eng. Comput. 51, 607–616. 10.1007/s11517-012-1031-4 23354828

[B22] Fok P. W. (2012). Mathematical model of intimal thickening in atherosclerosis: Vessel stenosis as a free boundary problem. J. Theor. Biol. 314, 23–33. 10.1016/j.jtbi.2012.07.029 22902428

[B23] Garikipati K. Arruda E. M. Grosh K. Narayanan H. Calve S. (2004). A continuum treatment of growth in biological tissue: The coupling of mass transport and mechanics. J. Mech. Phys. Solids 52, 1595–1625. 10.1016/j.jmps.2004.01.004

[B24] Gaziano T. Gaziano J. M. (2012). Chapter 1: global burden of cardiovascular disease, in Brunwald's Heart Disease: A Textbook of Cardiovascular Medicine, 9th Edn., eds Bonow R. Mann D. Zipes D. P. L.. (Philadelphia, PA: Elsevier).

[B25] Gijsen F. J. Wentzel J. J. Thury A. Mastik F. Schaar J. A. Schuurbiers J. C.. (2008). Strain distribution over plaques in human coronary arteries relates to shear stress. Am. J. Physiol. Heart Circ. Physiol. 295, 1608–1614. 10.1152/ajpheart.01081.2007 18621851

[B26] Goldstein J. L. Brown M. S. (1977). The low-density lipoprotein pathway and its relation to atherosclerosis. Anual Rev. Biochem. 46, 897–930. 10.1146/annurev.bi.46.070177.004341 197883

[B27] Guarino A. J. Tulenko T. N. Wrenn S. P. (2006). Sphingomyelinase-to-LDL molar ratio determines low density lipoprotein aggregation size: biological significance. Chem. Phys. Lipids 142, 33–42. 10.1016/j.chemphyslip.2006.02.020 16584719

[B28] Huang Y. Rumschitzki D. Chien S. Weinbaum S. (1994). A fiber matrix model for the growth of macromolecular leakage spots in the arterial intima. J. Biomech. Eng. 116, 430–445. 10.1115/1.2895794 7869719

[B29] Huang Z. J. Tarbell J. M. (1997). Numerical simulation of mass transfer in porous media of blood vessel walls. Am. J. Physiol. Heart Circ. Physiol. 273, 42-41. 10.1152/ajpheart.1997.273.1.H464 9249521

[B30] Humphrey J. D. (2002). Cardiovascular Solid Mechanics: Cells, Tissues, and Organs. Berlin: Springer.

[B31] Insull W. (2009). The pathology of atherosclerosis: plaque development and plaque responses to medical treatment. Am. J. Med. 122(1 Suppl.), S3–S14. 10.1016/j.amjmed.2008.10.013 19110086

[B32] Ivanova E. A. Myasoedova V. A. Melnichenko A. A. Grechko A. V. Orekhov A. N. (2017). Small dense low-density lipoprotein as biomarker for atherosclerotic diseases. Oxid. Med. Cell. Longev. 2017:1273042. 10.1155/2017/1273042 28572872 PMC5441126

[B33] Khan F. H. (2009). The Elements of Immunology. Delhi: Pearson Education.

[B34] Krombach F. Münzing S. Allmeling A. M. Gerlach J. T. Behr J. Dörger M. (1997). Cell size of alveolar macrophagues: an interspecies comparison. Environ Health Perspect 105, 1261–1263. 10.1289/ehp.97105s51261 9400735 PMC1470168

[B35] Levesque M. J. Liepsch D. Moravec S. Nerem R. M. (1986). Correlation of endothelial cell shape and wall shear stress in a stenosed dog aorta. Am. Heart Assoc. J. 6, 220–229. 10.1161/01.ATV.6.2.220 3954676

[B36] Lin S. J. Jan K. M. Weinbaum S. Chien S. (1989). Transendothelial transport of low density lipoprotein in association with cell mitosis in rat aorta. Arteriosclerosis 9, 230–236. 10.1161/01.ATV.9.2.230 2923579

[B37] Malek A. M. Alper S. L. (1999). Hemodynamic Shear Stress and Its Role in Atherosclerosis. Stress 282, 2035–2042. 10.1001/jama.282.21.2035 10591386

[B38] Malvè M. Chandra S. García A. Mena A. Martínez M. A. Finol E. A. Doblaré M. (2014). Impedance-based outflow boundary conditions for human carotid haemodynamics. Comput. Methods Biomech. Biomed. Eng. 17, 1248–1260. 10.1080/10255842.2012.744396 23387938

[B39] Martini F. H. (2012). Ch. 10: Muscle tissue, in Fundamentals of Anatomy and Physiology. Upper Saddle River, NY: Pearson Education.

[B40] Meyer G. Merval R. Tedgui A. (1996). Effects of pressure-induced stretch and convection on low-density lipoprotein and albumin uptake in the rabbit aortic wall. Circ. Res. 79, 532–540. 10.1161/01.RES.79.3.532 8781486

[B41] Milnor W. R. (1989). Hemodynamics. 2nd Edn. Baltimore, MD.

[B42] Morbiducci U. Mazzi V. Domanin M. Nisco G. D. Vergara C. Steinman D. A.. (2020). Wall shear stress topological skeleton independently predicts long-term restenosis after carotid bifurcation endarterectomy. Ann. Biomed. Eng. 48, 2936–2949. 10.1007/s10439-020-02607-9 32929560 PMC7723943

[B43] Ohayon J. Gharib A. M. Garcia A. Heroux J. Yazdani S. K. Malvè M.. (2011). Is arterial wall-strain stiffening an additional process responsible for atherosclerosis in coronary bifurcations?: an *in vivo* study based on dynamic CT and MRI. Am. J. Physiol. Heart Circ. Physiol. 301, H1097–H1106. 10.1152/ajpheart.01120.2010 21685261 PMC3191077

[B44] Olgac U. Kurtcuoglu V. Poulikakos D. (2008). Computational modeling of coupled blood-wall mass transport of LDL: Effects of local wall shear stress. Am. J. Physiol. Heart Circ. Physiol. 294, 909–919. 10.1152/ajpheart.01082.2007 18083898

[B45] Ougrinovskaia A. Thompson R. S. Myerscough M. R. (2010). An ODE model of early stages of atherosclerosis: Mechanisms of the inflammatory response. Bull. Math. Biol. 72, 1534–1561. 10.1007/s11538-010-9509-4 20440571

[B46] Peiffer V. Sherwin S. J. Weinberg P. D. (2013). Computation in the rabbit aorta of a new metric-the transverse wall shear stress-to quantify the multidirectional character of disturbed blood flow. J. Biomech. 46, 2651–2658. 10.1016/j.jbiomech.2013.08.003 24044966 PMC3807647

[B47] Perktold K. Resch M. Florian H. (1991). Pulsatile non-newtonian flow characteristics in a three-dimensional human carotid bifurcation model. J. Biomech. Eng. 113, 464–475. 10.1115/1.2895428 1762445

[B48] Prosi M. Zunino P. Perktold K. Quarteroni A. (2005). Mathematical and numerical models for transfer of low-density lipoproteins through the arterial walls: A new methodology for the model set up with applications to the study of disturbed lumenal flow. J. Biomech. 38, 903–917. 10.1016/j.jbiomech.2004.04.024 15713312

[B49] Sáez P. Malvè M. Martínez M. A. (2015). A theoretical model of the endothelial cell morphology due to different waveforms. J. Theor. Biol. 379, 16–23. 10.1016/j.jtbi.2015.04.038 25956359

[B50] Sáez P. Peña E. Ángel Martínez M. Kuhl E. (2013). Mathematical modeling of collagen turnover in biological tissue. J. Math. Biol. 67, 1765–1793. 10.1007/s00285-012-0613-y 23129392

[B51] Schwenke D. D. Carew T. E. (1989). Initiation of atherosclerotic lesions in cholesterol-fed rabbits, II: selective retention of LDL vs. selective increases in LDL permeability in susceptible sites of arteries. Arteriosclerosis 9:908–918. 10.1161/01.atv.9.6.908 2590068

[B52] Siogkas P. Sakellarios A. Exarchos T. P. Athanasiou L. Karvounis E. Stefanou K.. (2011). Multiscale-Patient-specific artery and atherogenesis models. IEEE Trans. Biomed. Eng. 58(12 Part 2), 3464–3468. 10.1109/TBME.2011.2164919 21846599

[B53] Sommer G. Regitnig P. Költringer L. Holzapfel G. A. (2010). Biaxial mechanical properties of intact and layer-dissected human carotid arteries at physiological and supraphysiological loadings. Am. J. Physiol. Heart Circ. Physiol. 298, 898–912. 10.1152/ajpheart.00378.2009 20035029

[B54] Steinberg D. Khoo J. C. Glass C. K. Palinski W. Almazan F. (1997). A new approach to determining the rates of recruitment of circulating leukocytes into tissues: Application to the measurement of leukocyte recruitment into atherosclerotic lesions. Proc. Natl. Acad. Sci. U.S.A. 94, 4040–4044. 10.1073/pnas.94.8.4040 9108101 PMC20564

[B55] Tedgui A. Lever M. J. (1984). Filtration through damaged and undamaged rabbit thoracic aorta. Am. J. Physiol. Heart Circ. 247, 784–791. 10.1152/ajpheart.1984.247.5.H784 6496759

[B56] Vargas C. B. Vargas F. F. Pribyl J. G. Blackshear P. L. (1979). Hydraulic conductivity of the endothelial and outer layers of the rabbit aorta. Am. J. Physiol. Heart Circ. 236, 56–60. 10.1152/ajpheart.1979.236.1.H53 434174

[B57] Weinbaum S. Tzeghai G. Ganatos P. Pfeffer R. Chien S. (1985). Effect of cell turnover and leaky junctions on arterial macromolecular transport. Am. Physiol. Soc. 248, 945–960. 10.1152/ajpheart.1985.248.6.H945 4003572

[B58] Younis H. F. Kaazempur-Mofrad M. R. Chan R. C. Isasi A. G. Hinton D. P. Chau A. H.. (2004). Hemodynamics and wall mechanics in human carotid bifurcation and its consequences for atherogenesis: Investigation of inter-individual variation. Biomech. Model. Mechanobiol. 3, 17–32. 10.1007/s10237-004-0046-7 15300454

[B59] Yuan F. Chien S. Weinbaum S. (1991). A new view of convective-diffusive transport processes in the arterial intima. J. Biomech. Eng. 113, 314–329. 10.1115/1.2894890 1921359

[B60] Zahedmanesh H. Van Oosterwyck H. Lally C. (2012). A multi-scale mechanobiological model of in-stent restenosis: deciphering the role of matrix metalloproteinase and extracellular matrix changes. Comp. Methods Biomech. Biomed. Eng. 17, 813–828. 10.1080/10255842.2012.716830 22967148

[B61] Zhao B. Li Y. Buono C. Waldo S. W. Jones N. L. Mori M.. (2006). Constitutive receptor-independent low density lipoprotein uptake and cholesterol accumulation by macrophages differentiated from human monocytes with macrophage-colony-stimulating factor (M-CSF). J. Biol. Chem. 281, 15757–15762. 10.1074/jbc.M510714200 16606620

[B62] Zhao S. Z. Ariff B. Long Q. Hughes A. D. Thom S. A. Stanton A. V.. (2002). Inter-individual variations in wall shear stress and mechanical stress distributions at the carotid artery bifurcation of healthy humans. J. Biomech. 35, 1367–1377. 10.1016/S0021-9290(02)00185-9 12231282

[B63] Zhao W. Oskeritzian C. A. Pozez A. L. Schwartz L. B. (2005). Cytokine production by skin-derived mast cells: endogenous proteases are responsible for degradation of cytokines. J. Immunol. 175, 2635–2642. 10.4049/jimmunol.175.4.2635 16081839

[B64] Zohdi T. I. Holzapfel G. A. Berger S. A. (2004). A phenomenological model for atherosclerotic plaque growth and rupture. J. Theor. Biol. 227, 437–443. 10.1016/j.jtbi.2003.11.025 15019510

