# Supplementary material for: Effects of the Haemodynamic Stimulus on the Location of Carotid Plaques Based on a Patient-Specific Mechanobiological Plaque Atheroma Formation Model
Source: Front Bioeng Biotechnol. 2021 Jun 14;9:690685. doi: 10.3389/fbioe.2021.690685 (PMC8236601; doi:10.3389/fbioe.2021.690685)
Supplement: Supplementary file 1 [file Data_Sheet_1.pdf]

Supplementary Material

1 SUPPLEMENTARY DATA

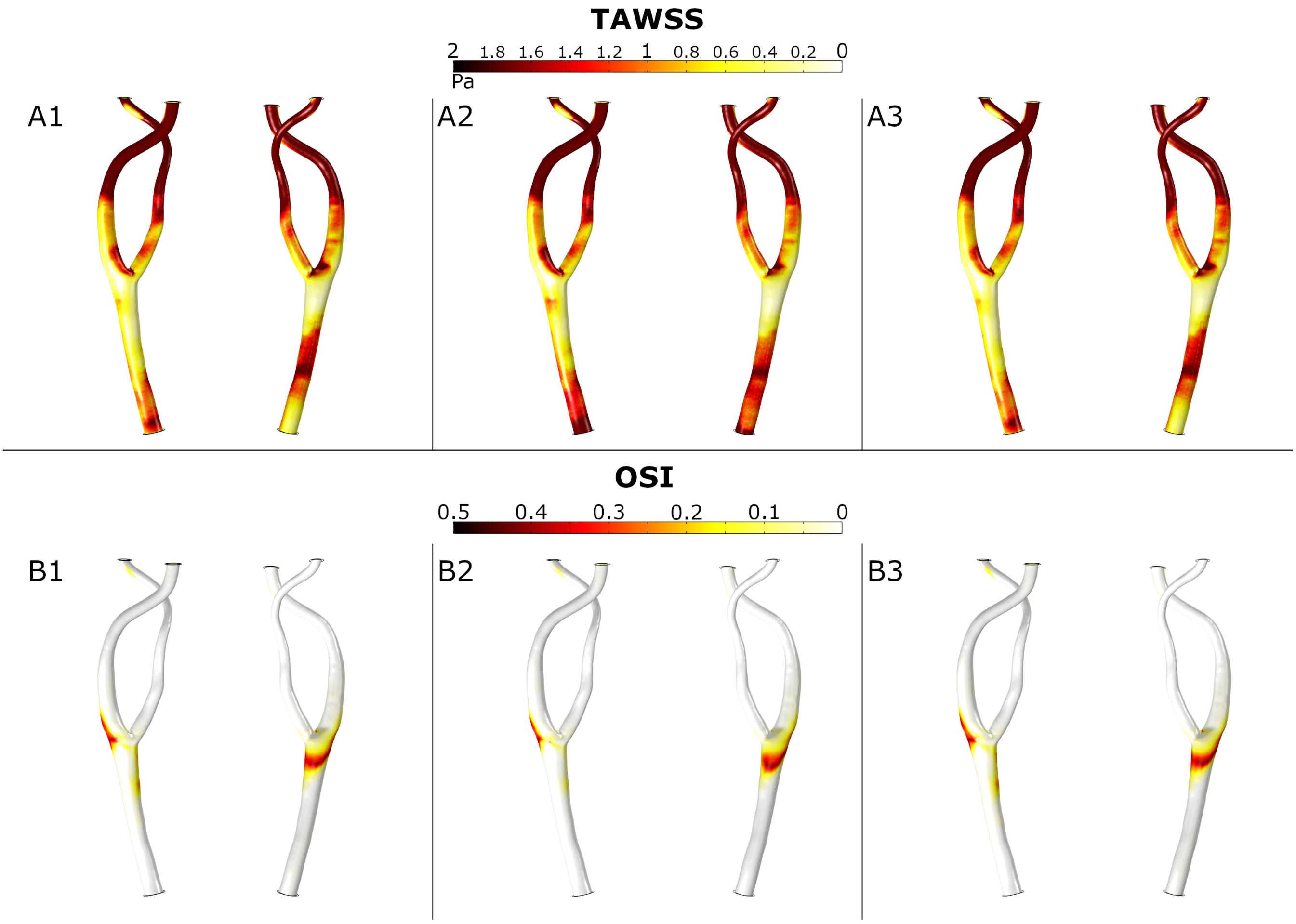

Figure S1. Mesh sensibility for the lumen of geometry ”A”, depending on the number of boundary layers. Letter ”A” corresponds to the distribution of TAWSS with a threshold of 2Pa while letter ”B” correlates with OSI. Numbers 1, 2 and 3 correspond to 2, 3 and 4 boundary layers, respectively.

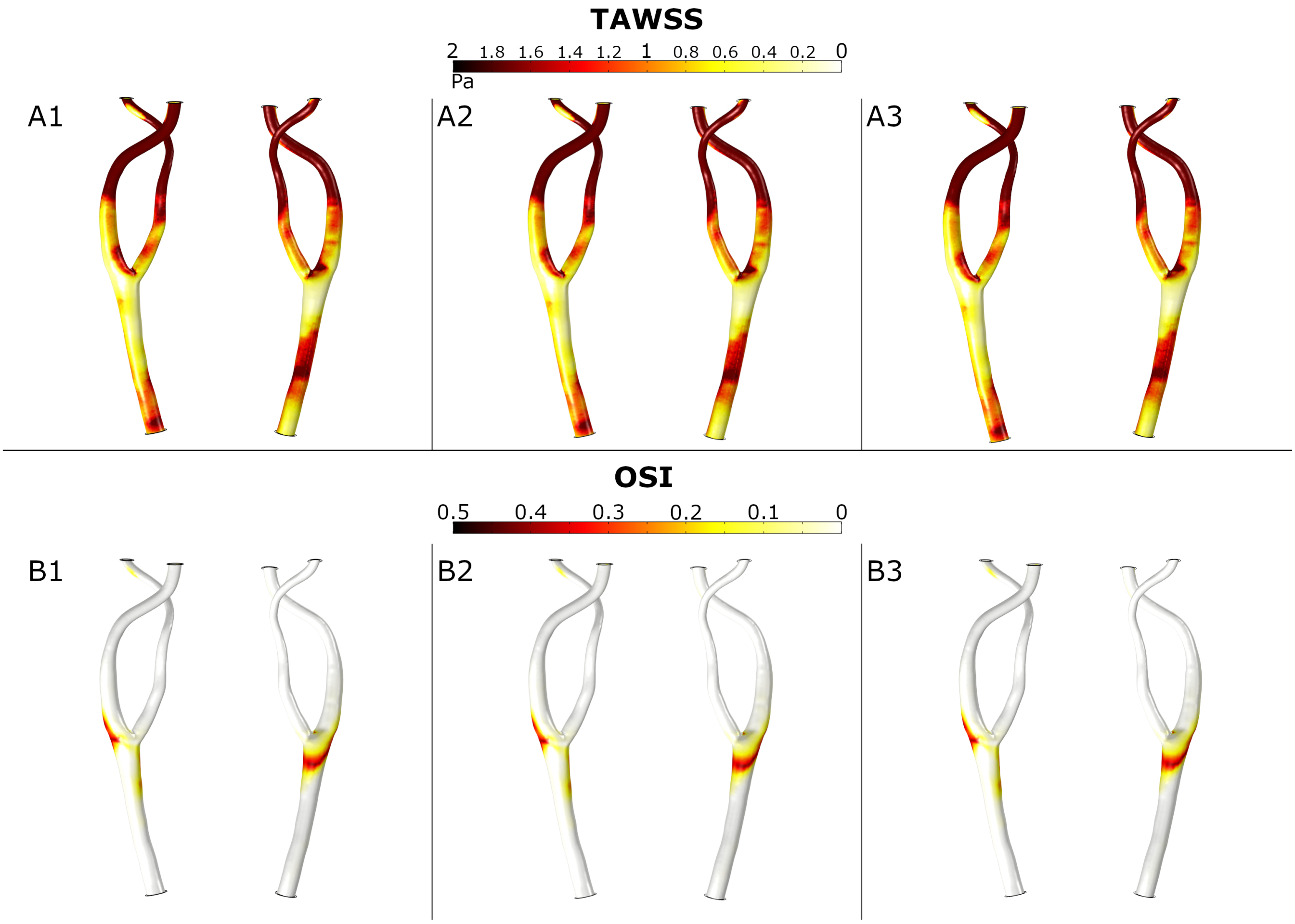

Figure S2. Mesh sensibility for the lumen of geometry ”A”, depending on the element size. Letter ”A” corresponds to the distribution of TAWSS with a threshold of 2Pa while letter ”B” correlates with OSI. Numbers 1, 2 and 3 correspond to 715620, 846077 and 1343776 elements, respectively.
